# Growth and Viability of Cutaneous Squamous Cell Carcinoma Cell Lines Display Different Sensitivities to Isoform-Specific Phosphoinositide 3-Kinase Inhibitors

**DOI:** 10.3390/ijms22073567

**Published:** 2021-03-30

**Authors:** Viviana Mannella, Kira Boehm, Suheyla Celik, Tasnim Ali, Amnah N. Mirza, Mariam El Hasnaouy, Andreas Kaffa, Yanshuang Lyu, Donya Kafaei Golahmadi, Irene M. Leigh, Daniele Bergamaschi, Catherine A. Harwood, Tania Maffucci

**Affiliations:** 1 Centre for Cell Biology and Cutaneous Research, Blizard Institute, Barts and The London School of Medicine and Dentistry, Queen Mary University of London, London E1 2AT, UK; viviana.mannella@gmail.com (V.M.); kiraboehm@ntlworld.com (K.B.); suheylacelik98@gmail.com (S.C.); tasnimali@protonmail.com (T.A.); amnahmirza9@gmail.com (A.N.M.); mariam.elhasnaouy@gmail.com (M.E.H.); andreaskaffa@hotmail.com (A.K.); yanshuang.lv@se17.qmul.ac.uk (Y.L.); donya94@hotmail.it (D.K.G.); d.bergamaschi@qmul.ac.uk (D.B.); catherine.harwood1@nhs.net (C.A.H.); 2 Department of Clinical Medicine, School of Queen Mary, Nanchang University, Nanchang 330006, China; 3 Institute of Dentistry, Barts and The London School of Medicine and Dentistry, Queen Mary University of London, London E1 2AD, UK; i.m.leigh@qmul.ac.uk

**Keywords:** cutaneous squamous cell carcinoma, phosphoinositide 3-kinases, mTOR, isoform-specific PI3K inhibitors, BYL719

## Abstract

Cutaneous squamous cell carcinomas (cSCCs) account for about 20% of keratinocyte carcinomas, the most common cancer in the UK. Therapeutic options for cSCC patients who develop metastasis are limited and a better understanding of the biochemical pathways involved in cSCC development/progression is crucial to identify novel therapeutic targets. Evidence indicates that the phosphoinositide 3-kinases (PI3Ks)/Akt pathway plays an important role, in particular in advanced cSCC. Questions remain of whether all four PI3K isoforms able to activate Akt are involved and whether selective inhibition of specific isoform(s) might represent a more targeted strategy. Here we determined the sensitivity of four patient-derived cSCC cell lines to isoform-specific PI3K inhibitors to start investigating their potential therapeutic value in cSCC. Parallel experiments were performed in immortalized keratinocyte cell lines. We observed that pan PI3Ks inhibition reduced the growth/viability of all tested cell lines, confirming the crucial role of this pathway. Selective inhibition of the PI3K isoform p110α reduced growth/viability of keratinocytes and of two cSCC cell lines while affecting the other two only slightly. Importantly, p110α inhibition reduced Akt phosphorylation in all cSCC cell lines. These data indicate that growth and viability of the investigated cSCC cells display differential sensitivity to isoform-specific PI3K inhibitors.

## 1. Introduction

Keratinocyte carcinomas (KCs) are the most common cancers in the UK, with over 210,000 tumors reported in 2015 [1]. KCs derive from epidermal keratinocytes, specialized cells that proliferate in the basal layer of the epidermis and differentiate as they migrate towards the surface of the skin where they form a tough keratinized layer known as the stratum corneum [2]. KCs are divided into basal cell carcinomas and cutaneous squamous cell carcinomas (cSCCs), with cSCC accounting for about 20% of KCs diagnoses [3]. Mortality rate of cSCC patients who develop metastasis is very high [4] because of the limited therapeutic options for this subset of patients, mainly due to a limited knowledge of the genetic drivers of metastasis [5]. Cemiplimab, a programmed death-1 receptor blocking antibody, belonging to the group of immune checkpoint inhibitors (ICI), was approved by the Food and Drug Administration in 2018 for patients with metastatic cSCC or locally advanced cSCC who are not candidates for curative surgery or curative radiation [6]. It remains the only approved systemic therapy, having been confirmed recently as the recommended first line treatment for these patients [6,7]. The use of ICI in organ transplant recipients and other patients on immunosuppressive medication, however, is still limited [6,8]. Furthermore, results from a phase I/II clinical trial of cemiplimab showed that the best overall response rate was 50% in the phase I and 48% in the phase II cohort [9]. A better understanding of the biochemical pathways involved in cSCC development and progression is crucial to identify novel potential therapeutic targets.

Phosphoinositide 3-kinases (PI3Ks) are a family of lipid kinases that catalyze the phosphorylation of the 3′ position within the inositol ring of distinct phosphoinositides [10,11]. Eight mammalian PI3Ks exist, grouped into three classes [12], and they regulate activation of several proteins through synthesis of specific lipid products, ultimately controlling many cellular functions, including proliferation, survival, migration and metabolism [13,14]. The isoforms belonging to the class I subgroup, are solely responsible for the synthesis of phosphatidylinositol 3,4,5-trisphosphate [PtdIns(3,4,5)*P_3_*] in vivo and are dimers of a regulatory and one of four catalytic subunits, namely p110α, p110β, p110δ and p110γ [12]. The best characterized amongst all proteins activated by class I PI3Ks is the Serine/Threonine kinase Akt [15], whose mechanism of activation involves its translocation to the plasma membrane, induced by binding of its pleckstrin homology domain to PtdIns(3,4,5)*P_3_*, and subsequent phosphorylation at its residues Thr308 and Ser473. Once activated, Akt regulates a plethora of proteins, including mechanistic (or mammalian) target of rapamycin (mTOR), a master kinase that controls several cellular functions, such as protein synthesis, ribosome biogenesis, cell growth and autophagy [16,17]. The pathway is negatively regulated by the tumor suppressor Phosphatase and tensin homolog (PTEN) that dephosphorylates PtdIns(3,4,5)*P_3_* thus preventing Akt activation [18].

The PI3K/Akt/mTOR signaling pathway is important for epidermal homeostasis [19,20,21], as strongly indicated by a number of severe disorders known as PTEN hamartoma tumor syndromes which are caused by germline mutations of *PTEN* and are characterized by hyperplastic changes in the skin [22]. Additional lines of evidence include data from animal models, such as mice bearing a keratinocyte-specific *PTEN* null mutation which are characterized by epidermal hyperplasia and hyperkeratosis [23], mice with ablation of two of the three Akt isoforms, Akt1 and Akt2, which lack the stratum corneum and die neonatally, possibly because of defects in the skin barrier [24] and mice carrying an epidermis-specific deletion of *mTOR* which die shortly after birth due to impaired early epidermal differentiation and lack of a protective barrier development [25].

Consistent with its role in epidermal homeostasis, evidence also suggests that the PI3K/Akt/mTOR pathway is important during cSCC development and progression [26]. Activation of a number of key proteins involved in this pathway was detected in human cSCC by reverse phase protein microarray analysis [27]. Phosphorylation of Akt, mTOR and downstream effectors was also observed more frequently in malignant compared to benign epidermal tumors or normal skin [28]. In addition, a study showed upregulation of Akt2, with parallel downregulation of Akt1, in cSCC, with high Akt2 expression and high levels of phosphorylated Akt observed in all examined poorly differentiated cSCC [29]. The importance of this pathways in skin tumorigenesis is further supported by results from in vivo studies. Specifically, it was reported that mice bearing a keratinocyte-specific *PTEN* null mutation developed tumors more rapidly upon chemical treatment as well as spontaneous tumors, mostly squamous papillomas, many of which progressed into invasive cSCC [23]. Additional studies demonstrated that Akt activity was increased in the two-stage model of mouse skin carcinogenesis [30], and that the enzyme had a key role in promoting insulin like growth factor-1-mediated mouse skin cancer [31,32]. Consistent with this, transgenic mice expressing increased levels of Akt or constitutively active Akt in the basal layer were more sensitive to two-stage skin carcinogenesis and to the tumor promoter 12-*O*-tetradecanoylphorbol-13-acetate [33] and overexpression of Akt in keratinocytes accelerated tumorigenesis in vivo [31].

While the importance of the PI3K/Akt/mTOR pathway is supported by several data, much less is known about the specific contribution of each distinct class I PI3K isoform to cSCC. Moreover, whether the use of available isoform-specific inhibitors might represent a potential therapeutic strategy in cSCC remains to be clarified. Recently, an extensive characterization of a panel of patient-derived cSCC cell lines has been reported, validating their use for preclinical testing [34]. Here, we investigated the sensitivity of some of these cells to selective class I PI3Ks inhibitors to identify the main isoform(s) involved in regulation of their growth, viability and Akt activation, and to start evaluating the potential therapeutic value of these inhibitors in cSCC.

## 2. Results

### 2.1. Expression of Class I PI3K Isoforms in Keratinocytes and cSCC Cell Lines

**Figure 1:**
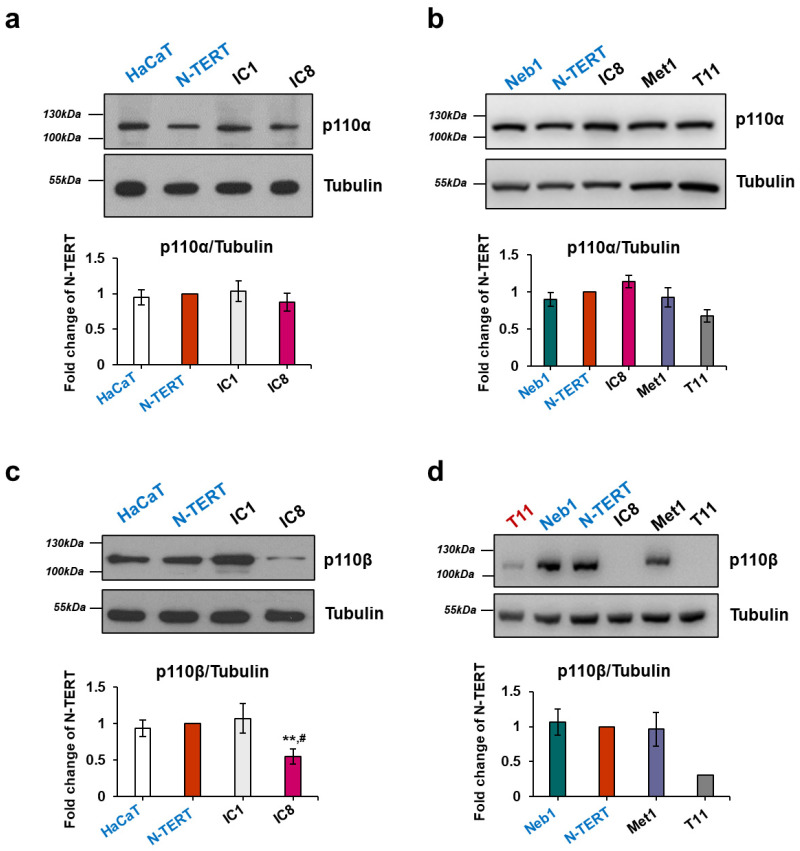
Keratinocyte and cutaneous squamous cell carcinoma (cSCC) cell lines express class I phosphoinositide 3-kinase (PI3K) isoforms p110α and p110β. Immortalized keratinocytes (HaCaT, N-TERT and Neb1, labelled in blue) and cSCC cells (IC1, IC8, Met1 and T11) were lysed with 2% SDS and expression of the indicated class I PI3K isoforms was analyzed by Western blotting. Tubulin was used as loading control. Signals were visualized using X-ray films and a film processor (**a**,**c**) or using Chemidoc™ MP Imaging System (**b**,**d**). Representative blots are shown. Graphs indicate data from densitometry analysis for each enzyme, normalized to Tubulin and expressed as fold change of results from N-TERT cells. Data are means ± SEM from the following numbers of lysates, prepared independently from different batches of cells: n = 6 ((**a**), apart from IC1, n = 5), n = 3 (**b**), n = 6 (**c**) and n = 3 ((**d**), apart from T11, n = 1). Note that p110β was barely detectable and not quantifiable in IC8 cells using Chemidoc™ MP Imaging System (**d**) therefore densitometry analysis could not be performed for these cells in these experiments. Similarly, the band was detectable and quantifiable in only one of the T11 lysates in these conditions therefore only this set (labelled in red) was used for analysis in (**d**). See also Supplementary Figure S1d. ** *p* < 0.01 vs. N-TERT, ^#^ *p* < 0.05 vs. HaCaT.

Several lines of evidence indicate that the PI3K/Akt/mTOR pathway is critical for keratinocytes [19,20,21] and cSCC [26,27,28,29,30,31,32,33]. In order to assess the potential therapeutic value of targeted inhibition of this pathway, we first determined the expression profile of class I PI3Ks in a panel of recently characterized cSCC cell lines [34,35] compared to immortalized keratinocyte cell lines. Lysates were analyzed by Western blotting and signals were visualized by using either a film processor (Figure 1a,c; Supplementary Figure S1a–c) or an imaging system (Figure 1b,d; Supplementary Figure S1d), as specified in the Methods. Attention was focused on class I isoforms as these enzymes are mainly responsible for Akt/mTOR activation in vivo. Data confirmed endogenous expression of the class I isoforms p110α and p110β in the immortalized keratinocytes HaCaT (Figure 1a,c), N-TERT (Figure 1; Supplementary Figure S1a,b,d) and Neb1 (Figure 1b,d; Supplementary Figure S1d), consistent with previous data reporting expression of these enzymes in keratinocytes [20]. Expression of p110α was also detected in a panel of cSCC cell lines, namely IC1 (Figure 1a; Supplementary Figure S1a), IC8 (Figure 1a,b; Supplementary Figure S1a), Met1 (Figure 1b; Supplementary Figure S1a), T11 (Figure 1b) and T1 (Supplementary Figure S1a). Moreover, densitometry analysis indicated that expression levels of p110α were similar in all tested cell lines, although possibly slightly reduced in T11 cells compared to N-TERT (Figure 1b). In contrast to p110α, expression of p110β varied between the cSCC cells, with IC8 (Figure 1c,d; Supplementary Figure S1b,d), T11 (Figure 1d; Supplementary Figure S1d) and T1 (Supplementary Figure S1b) displaying much lower levels of the enzyme compared to the other cSCC cells and to the immortalized keratinocytes. No major differences in the levels of either Tubulin or Glyceraldehyde 3-phosphate dehydrogenase (GAPDH) was observed between the cell lines (Supplementary Figure S1c) and similar results were obtained when levels of p110β were normalized to either Tubulin (Figure 1d) or GAPDH (Supplementary Figure S1d), validating their use as loading control in these experiments.

Expression of p110γ and p110δ was also observed (Supplementary Figure S2). These data confirmed that class I PI3K isoforms are detectable in keratinocytes and cSCC cells. Based on these results, we focused our investigation on the cSCC cell lines Met1 and IC1, which express similar levels of p110α and p110β compared to the immortalized keratinocytes, and on IC8 and T11 cells which express comparable (IC8) or slightly lower (T11) levels of p110α and very reduced levels of p110β compared to the immortalized keratinocytes.

### 2.2. Effect of PI3K/Akt/mTOR Pathway Inhibition on Keratinocytes and cSCC Cell Lines

**Figure 2:**
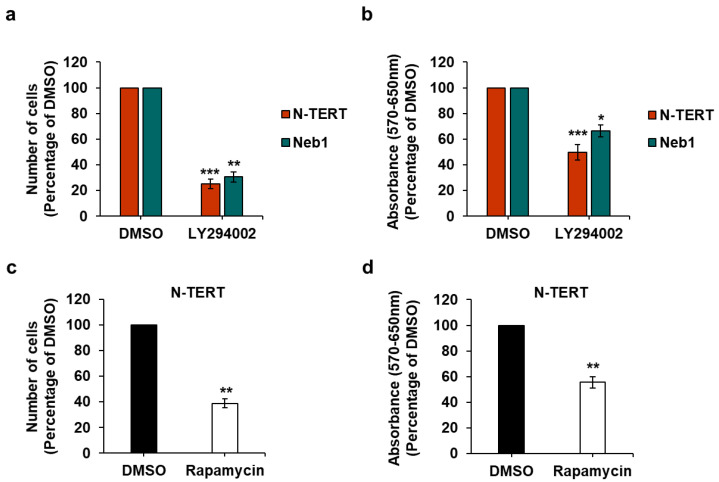
The PI3K/Akt/mTOR pathway regulates growth and viability of keratinocytes. (**a**,**b**) N-TERT and Neb1 cells were treated with 10 µM LY294002 or vehicle alone (DMSO) in complete medium supplemented with 10% fetal bovine serum (FBS). (**c**,**d**) N-TERT cells were treated with 10 nM rapamycin in complete medium supplemented with 10% FBS. After 72 h, number of cells was assessed by cell counting (**a**,**c**) and cell viability was assessed by MTT assays (**b**,**d**). Data are expressed as percentage of results from cells treated with DMSO and are means ± SEM from n = 6 ((**a**,**b**), N-TERT), n = 3 ((**a**,**b**), Neb1), n = 3 (**c**) and n = 4 (**d**) independent experiments performed in duplicate (**a**,**c**) or triplicate (**b**,**d**). * *p* < 0.05, ** *p* < 0.01, *** *p* < 0.001 vs. DMSO.

Having confirmed expression of class I PI3K isoforms, we next determined whether these enzymes were involved in regulation of growth and viability of the selected cell lines. First, we observed that treatment with the pan PI3K inhibitor LY294002 reduced the number of N-TERT and Neb1 cells (Figure 2a) as well as their viability assessed by MTT assays (Figure 2b). Treatment with the mTOR inhibitor rapamycin also reduced numbers (Figure 2c) and viability (Figure 2d) of N-TERT cells, consistent with data in the literature indicating the crucial role of the PI3K/Akt/mTOR pathway in keratinocytes [19,20,21].

**Figure 3:**
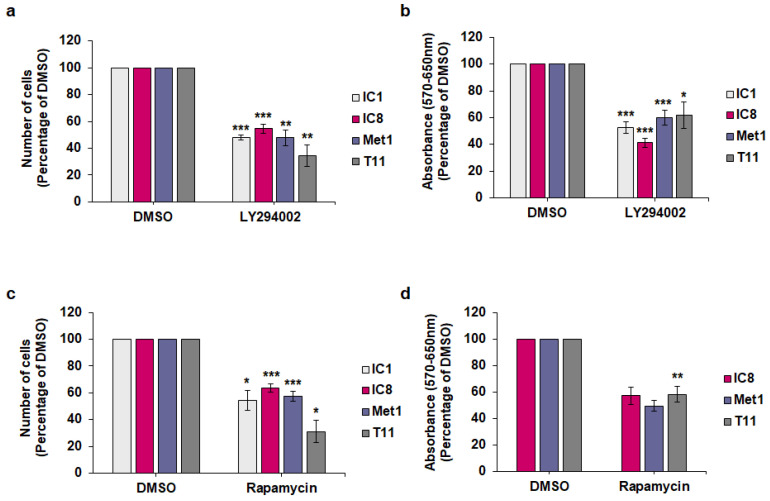
The PI3K/Akt/mTOR pathway regulates growth and viability of cSCC cell lines. The indicated cSCC cell lines were treated with 10 µM LY294002 (**a**,**b**) or 10 nM rapamycin (**c**,**d**) in complete medium supplemented with 10% FBS. Control cells were treated with vehicle alone (DMSO). After 72 h, number of cells was assessed by cell counting (**a**,**c**) and cell viability was assessed by MTT assays (**b**,**d**). Data are expressed as percentage of results from control cells and are means ± SEM from n = 4 ((**a**), IC1, IC8, Met1), n = 7 ((**a**), IC1); n = 5 ((**b**), IC8), n = 7 ((**b**), Met1), n = 4 ((**b**), T11), n = 10 ((**b**), IC1); n = 3 ((**c**), IC1,T11), n = 6 ((**c**), Met1), n = 9 ((**c**), IC8); n = 2 ((**d**), IC8, Met1) and n = 3 ((**d**), T11) independent experiments performed in duplicate (**a**,**c**) or triplicate (**b**,**d**). * *p* < 0.05, ** *p* < 0.01, *** *p* < 0.001 vs. DMSO.

Next, we tested the effect of these inhibitors on the selected cSCC cell lines. Data showed that treatment with LY294002 (10 µM) reduced number (Figure 3a) and viability (Figure 3b) of all selected cSCC cells. Inhibition of growth (Supplementary Figure S3a) and viability (Supplementary Figure S3b) was also detected upon treatment of IC8 and Met1 cells with lower concentrations of LY294002. Similarly, treatment with 10 nM rapamycin reduced the number of all selected cSCC cell lines (Figure 3c) as well as viability of IC8, Met1 and T11 cells (Figure 3d). More specifically, rapamycin reduced numbers of IC8 and Met1 cells in a dose dependent manner (Supplementary Figure S3c). Finally, treatment with 50 nM rapamycin reduced viability of IC1 as well as IC8 and Met1 cells (Supplementary Figure S3d). Treatment with vehicle alone did not affect numbers and viability of N-TERT (Supplementary Figure S4a,b), Neb1 (Supplementary Figure S4a,b) and IC8 (Supplementary Figure S4c,d) cells or viability of Met1 and IC1 cells (Supplementary Figure S4d).

Taken together, these data confirmed that the PI3K/Akt/mTOR pathway plays a key role in keratinocytes and in cSCC cell growth and viability.

### 2.3. Effect of Isoform-Specific PI3K Inhibitors on Keratinocytes Growth and Viability

**Figure 4:**
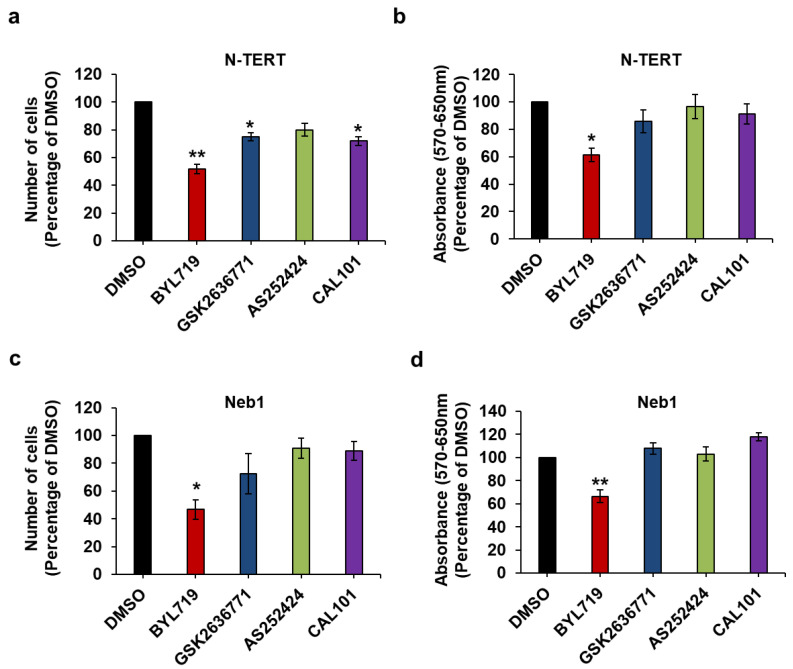
The p110α inhibitor BYL719 is the most effective in reducing both cell numbers and viability of keratinocytes. N-TERT (**a**,**b**) and Neb1 (**c**,**d**) cells were treated with 1 µM of the indicated inhibitors or vehicle (DMSO) in complete medium supplemented with 10% FBS. After 72 h, number of cells was assessed by cell counting (**a**,**c**) and cell viability was assessed by MTT assays (**b**,**d**). Data are expressed as percentage of results from cells treated with DMSO and are means ± SEM from n = 3 (**a**), n = 3 (**b**), n = 3 (**c**) and n = 4 (**d**) independent experiments performed in duplicate (**a**,**c**) or triplicate (**b**,**d**). * *p* < 0.05, ** *p* < 0.01 vs. DMSO.

To identify the main class I PI3K isoform(s) involved in regulation of keratinocytes and cSCC growth and viability, cells were treated with isoform-specific PI3K inhibitors, namely BYL719 (targeting p110α primarily), GSK2636771 (targeting p110β primarily), AS252424 (targeting p110γ primarily) and CAL101 (targeting p110δ primarily). First, investigation of the effect of these inhibitors on immortalized keratinocytes revealed that BYL719 reduced both cell numbers (Figure 4a,c) and viability (Figure 4b,d) of N-TERT and Neb1 cells. At the tested concentration, none of the other inhibitors had an effect, apart from GSK2636771 and CAL101 reducing N-TERT cell numbers significantly but slightly (Figure 4a).

As a slight reduction in cell numbers was observed upon treatment with the p110β inhibitor GSK2636771 in N-TERT cells (Figure 4a), we next compared the effects of p110α and p110β inhibition by performing a dose response analysis of the effect of BYL719 and GSK2636771. Data confirmed that BYL719 had the strongest inhibitory effect on N-TERT cell numbers (Supplementary Figure S5a) and viability (Supplementary Figure S5b) at the tested concentrations. Similar results were obtained by assessing the effect of the inhibitors on HaCaT cell numbers (Supplementary Figure S5c). As observed for the other cell lines used in this study, no effect on cell numbers was detected in untreated HaCaT compared to cells treated with vehicle alone (Supplementary Figure S5d).

**Figure 5:**
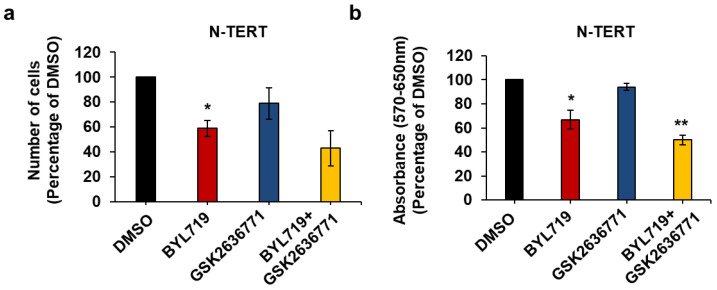
p110β does not play a major role in N-TERT growth and viability upon p110α inhibition. N-TERT cells were treated with 1 µM of the indicated inhibitors alone or in combination in complete medium supplemented with 10% FBS. Control cells were treated with DMSO. After 72 h, number of cells was assessed by cell counting (**a**) and cell viability was assessed by MTT assays (**b**). Data are expressed as percentage of results from control cells and are means ± SEM from n = 3 (**a**) and n = 4 (**b**) independent experiments performed in duplicate (**a**) or triplicate (**b**). * *p* < 0.05, ** *p* < 0.01 vs. DMSO.

Treatment with BYL719 appeared to reduce cell numbers and viability less than pan PI3K inhibition (Figure 2a,b); therefore, we next investigated whether p110β could have any compensatory role upon p110α inhibition, as observed in some cancer settings [36,37]. To this end, N-TERT cells were treated with BYL719 and GSK2636771 alone or in combination. Combination of the two compounds did not increase the inhibitory effect of BYL719 on N-TERT cell numbers (Figure 5a) and viability (Figure 5b) significantly, compared to treatment with BYL719 alone.

Taken together, these data demonstrated that BYL719 inhibits growth and viability of keratinocytes more potently than the other inhibitors tested, suggesting that p110α is the main PI3K isoform regulating such cellular functions in these cells.

### 2.4. Effect of Isoform-Specific PI3K Inhibitors on cSCC Cells Growth and Viability

**Figure 6:**
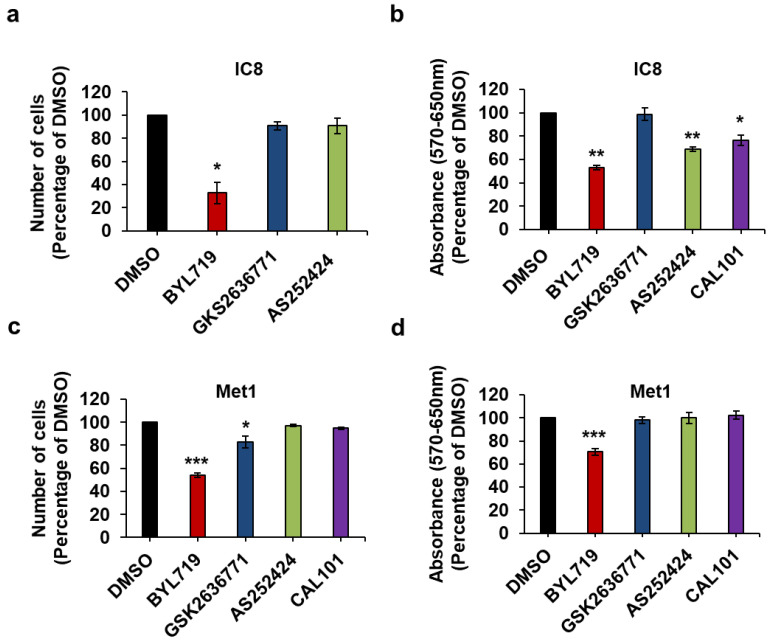
The p110α inhibitor BYL719 is the most effective in reducing IC8 and Met1 cell numbers and viability. IC8 (**a**,**b**) and Met1 (**c**,**d**) cells were treated with 1 µM of the indicated inhibitors or vehicle (DMSO) in complete medium supplemented with 10% FBS. After 72 h, number of cells was assessed by cell counting (**a**,**c**) and cell viability was assessed by MTT assays (**b**,**d**). Data are expressed as percentage of results from cells treated with DMSO and are means ± SEM from n = 3 ((**a**), apart from AS252424, n = 6), n = 3 (**b**), n = 5 ((**c**), apart from GSK2636771, n = 4) and n = 5 (**d**) independent experiments performed in duplicate (**a,c**) or triplicate (**b**,**d**). * *p* < 0.05, ** *p* < 0.01, *** *p* < 0.001 vs. DMSO.

Next, we assessed the effect of the inhibitors on the selected cSCC cell lines. We observed that the p110α inhibitor BYL719 clearly reduced the number (Figure 6a,c) and viability (Figure 6b,d) of IC8 and Met1 cells. Treatment of IC8 cells with AS252424 or CAL101 had a significant effect in the MTT assays (Figure 6b) although AS252424 did not seem to reduce numbers of these cells (Figure 6a). A slight reduction in the number of Met1 cells was also detected upon treatment with GSK2636771 (Figure 6c). Dose response analysis, however, confirmed a stronger inhibitory effect of BYL719 compared to GSK2636771 on both IC8 (Supplementary Figure S6a) and Met1 (Supplementary Figure S6c) cell numbers as well as IC8 viability (Supplementary Figure S6b). No effect on cell counting or MTT assays was observed in Met1 treated with AS252424 or CAL101 at the tested concentration (Figure 6c,d).

**Figure 7:**
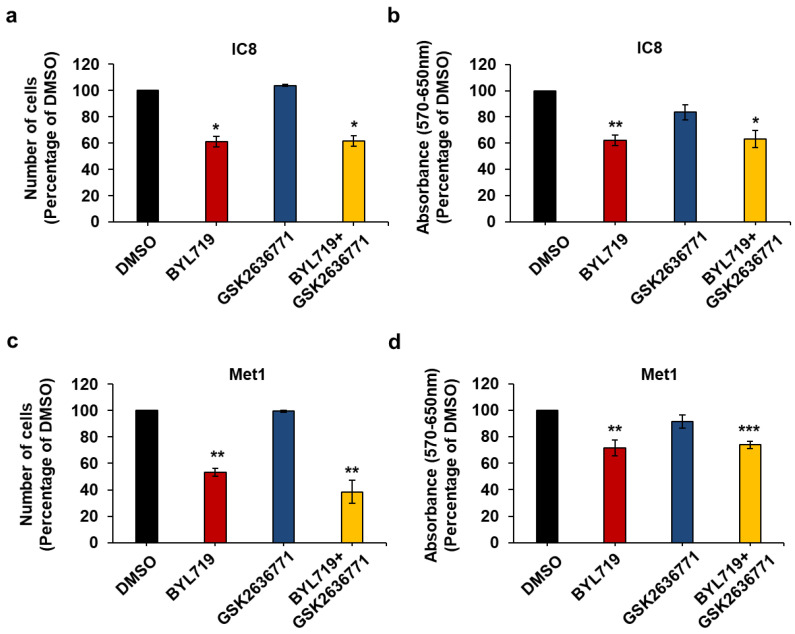
p110β does not contribute to IC8 and Met1 cell growth and viability upon p110α inhibition. IC8 (**a**,**b**) and Met1 (**c**,**d**) cells were treated with 1 µM of the indicated inhibitors alone or in combination in complete medium supplemented with 10% FBS. Control cells were treated with DMSO. After 72 h, number of cells was assessed by cell counting (**a**,**c**) and cell viability was assessed by MTT assays (**b**,**d**). Data are expressed as percentage of results from control cells and are means ± SEM (apart from (**c**), means ± SD) from n = 3 (**a**), n = 4 (**b**), n = 3 ((**c**), apart from GSK2636771, n = 2) and n = 6 (**d**) independent experiments performed in duplicate (**a**,**d**) or triplicate (**b**,**d**). * *p* < 0.05, ** *p* < 0.01, *** *p* < 0.001 vs. DMSO.

In addition, combination of BYL719 and GSK2636771 did not result in any significant increase in the effect of the p110α inhibitor on IC8 and Met1 cell numbers (Figure 7a,c) or viability (Figure 7b,d) compared to treatment with BYL719 alone.

Taken together these data indicate that IC8 and Met1 cells retain the same sensitivity to PI3K inhibition as normal keratinocytes and suggest that p110α is the main PI3K regulating growth and viability of these cSCC cell lines.

**Figure 8:**
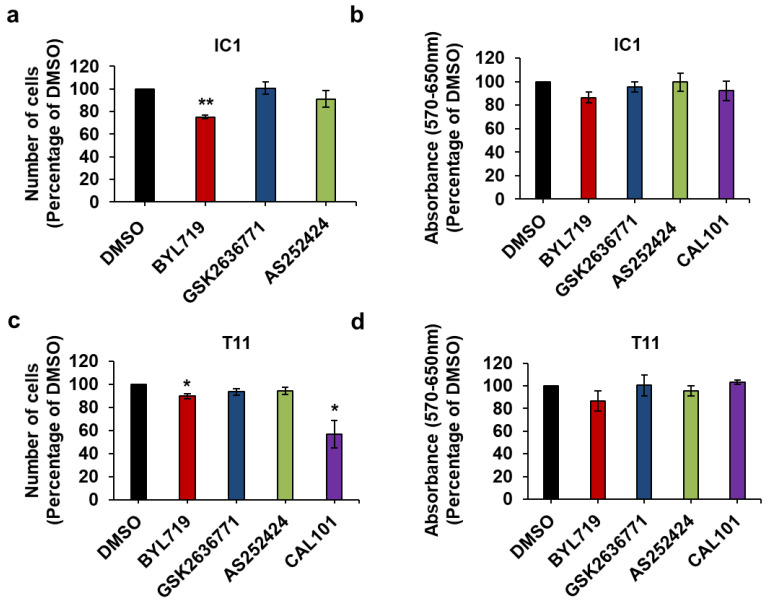
IC1 and T11 cells are more resistant to treatment with isoform-specific PI3K inhibitors than IC8 and Met1 cells. IC1 (**a**,**b**) and T11 (**c**,**d**) cells were treated with 1 µM of the indicated inhibitors or vehicle (DMSO) in complete medium supplemented with 10% FBS. After 72 h, number of cells was assessed by cell counting (**a**,**c**) and cell viability was assessed by MTT assays (**b**,**d**). Data are expressed as percentage of results from cells treated with DMSO and are means ± SEM from n = 3 ((**a**), apart from AS252424, n = 4), n = 4 ((**b**), apart from CAL101 n = 3), n = 4 ((**c**), apart from CAL101 n = 5) and n = 4 ((**d**)**,** apart from AS252424 and CAL101 n = 3) independent experiments performed in duplicate (**a**,**c**) or triplicate (**b**,**d**). * *p* < 0.05, ** *p* < 0.01 vs. DMSO.

In contrast to IC8 and Met1 cells, BYL719 had only a very small effect on IC1 (Figure 8a) and T11 (Figure 8c) cell numbers with reduction not reaching statistical significance in MTT assays (Figure 8b,d). No effect was detected in MTT assays upon treatment of IC1 cells with lower concentrations of either BYL719 or GSK2636771 (Supplementary Figure S6d). None of the other inhibitors affected IC1 or T11 cells at the concentration tested, apart from CAL101 reducing T11 cell numbers significantly (Figure 8c).

Combined results from cell counting and MTT assays indicate that IC1 and T11 cells are more resistant to PI3K inhibition, in particular to p110α inhibition, compared to IC8 and Met1 cells.

**Figure 9:**
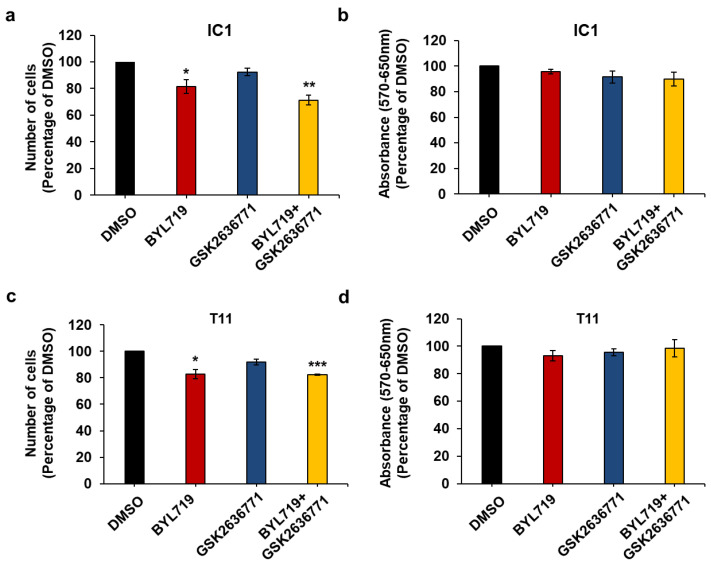
p110β does not compensate for p110α inhibition in IC1 and T11 cells. IC1 (**a**,**b**) and T11 (**c**,**d**) cells were treated with 1 µM of the indicated inhibitors alone or in combination in complete medium supplemented with 10% FBS. Control cells were treated with DMSO. After 72 h, number of cells was assessed by cell counting (**a**,**c**) and cell viability was assessed by MTT assays (**b**,**d**). Data are expressed as percentage of results from control cells and are means ± SEM from n = 4 (**a**), n = 7 (**b**) and n = 3 (**c**,**d**) independent experiments performed in duplicate (**a**,**c**) or triplicate (**b**,**d**). * *p* < 0.05, ** *p* < 0.01, *** *p* < 0.001.

To determine whether resistance to p110α inhibition was due to a compensatory role of p110β, we next investigated the effect of simultaneous inhibition of p110α and p110β on these cells. Consistent with our previous data, treatment with BYL719 alone reduced IC1 (Figure 9a) and T11 (Figure 9c) cell numbers only slightly and it did not have a detectable significant effect on MTT assays in either cell line (Figure 9b,d). Importantly, no further reduction in cell numbers (Figure 9a,c) and no effect on viability (Figure 9b,d) were observed when BYL719 was combined with GSK2636771.

These data indicate that reduced response to p110α inhibition in IC1 and T11 cells is not due to a compensatory mechanism dependent on p110β, as detected in some breast cancers [36,37]. Taken together, these data reveal a different sensitivity to PI3K inhibitors across a panel of cSCC cell lines.

### 2.5. Effect of Isoform-Specific PI3K Inhibitors on Akt Activation in cSCC Cells

**Figure 10:**
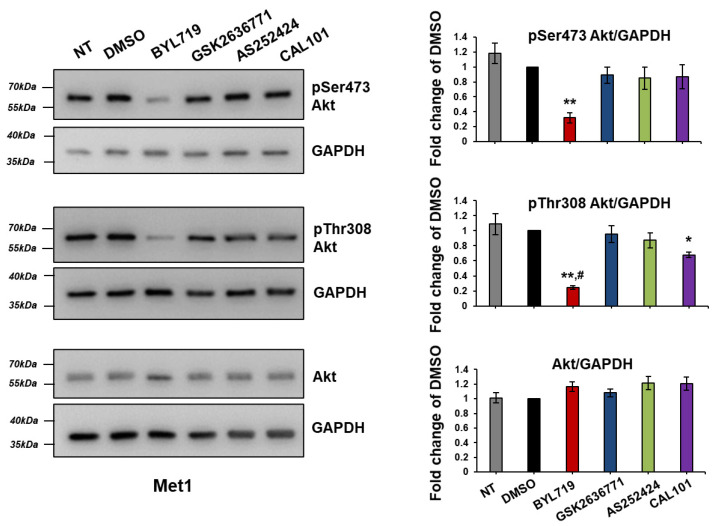
BYL719 inhibits Akt phosphorylation in Met1 cells. Met1 cells were treated with 1 µM of the isoform-specific inhibitors in complete medium supplemented with 10% FBS. Control cells were treated with DMSO or left untreated (NT). Cells were lysed after 1 h and Akt phosphorylation at residues Ser473 and Thr308 as well as total levels of Akt were determined by Western blotting. Glyceraldehyde 3-phosphate dehydrogenase (GAPDH) was used as loading control. Signals were visualized using Chemidoc™ MP Imaging System. Graphs indicate results from densitometry analysis of phosphorylated or total Akt normalized to loading control and expressed as fold change of results from DMSO-treated cells. Data are means ± SEM from n = 3 independent experiments (apart from NT in pSer437 Akt graph, n = 2). * *p* < 0.05, ** *p* < 0.01 vs. DMSO; ^#^ *p* < 0.05 vs. NT.

In order to determine whether the limited response to isoform-specific inhibitors observed in IC1 and T11 cells was due to an intrinsic resistance to PI3K inhibition, we next assessed the effect of these compounds on Akt activation in the selected cSCC cell lines. Treatment with BYL719 for 1 h strongly inhibited Akt phosphorylation at both Ser473 and Thr308 residues in Met1 cells without affecting the levels of total Akt, as determined by Western blotting followed by visualization using either Chemidoc™ MP Imaging system (Figure 10) or a film processor (Supplementary Figure S7). Similar results were obtained in IC8 cells (Supplementary Figure S8). No inhibition of Akt phosphorylation was detected when cells were treated with the other inhibitors at the tested concentration, apart from a slight reduction of Thr308 phosphorylation in Met1 cells upon treatment with CAL101 (Figure 10, Supplementary Figure S7).

**Figure 11:**
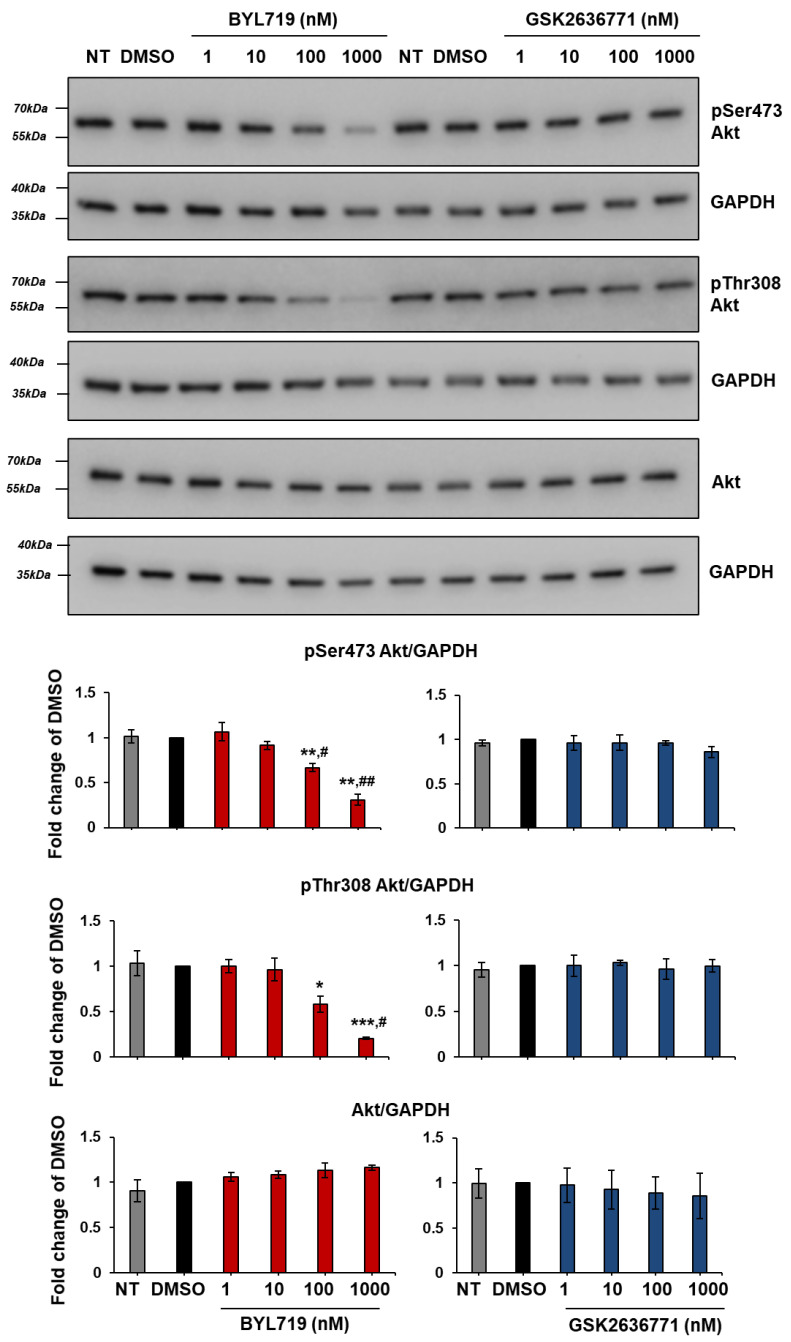
BYL719 inhibits Akt phosphorylation in Met1 cells in a dose-dependent manner. Met1 cells were treated with the indicated concentrations of BYL719 and GSK2636771 in complete medium supplemented with 10% FBS. Control cells were treated with DMSO or left untreated (NT). After 1 h, cells were Lysed and lysates were analyzed by Western blotting. GAPDH was used as loading control. Signals were visualized using Chemidoc™ MP Imaging System. Graphs indicate results from densitometry analysis of phosphorylated or total Akt normalized to loading control and expressed as fold change of results from DMSO-treated cells. Data are means ± SEM from n = 3 independent experiments (apart from data from cells treated with BYL719 1 nM, 10 nM and 100 nM, n = 4) * *p* < 0.05, ** *p* < 0.01, *** *p* < 0.001 vs. DMSO; ^#^ *p* < 0.05, ^##^ *p* < 0.01 vs. NT.

**Figure 12:**
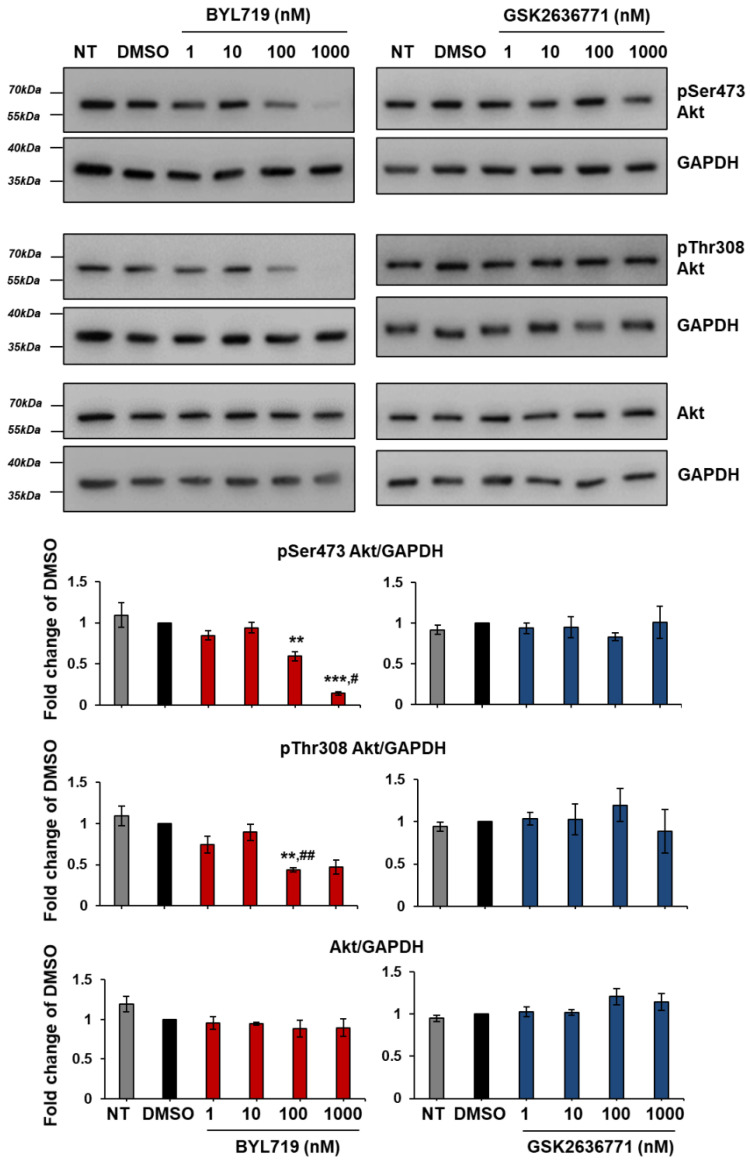
BYL719 inhibits Akt phosphorylation in IC8 cells in a dose-dependent manner. IC8 cells were left untreated (NT) or treated for 1 h with increasing concentrations of BYL719 and GSK2636771 in complete medium supplemented with 10% FBS. Control cells were treated with DMSO. Phosphorylation status and total levels of Akt were analyzed by Western blotting. GAPDH was used as loading control. Signals were visualized using Chemidoc™ MP Imaging System. Graphs show results from densitometry analysis of phosphorylated or total Akt normalized to loading control and expressed as fold change of results from DMSO-treated cells. Only blots showing clearly detectable bands were used for densitometry analysis. Data are means ± SEM from the following numbers of independent experiments: pSer473 Akt, n = 4 (apart from NT in BYL719 graph and from BYL719 1 nM, BYL719 10 nM and GSK2636771 100 nM, n = 3); pThr308 Akt, n = 4 (apart from BYL719 1 nM, 10 nM and 100 nM, n = 3 and BYL719 1 µM, n = 2); Akt, n = 4 (apart from NT, n = 3); ** *p* < 0.01, *** *p* < 0.001 vs. DMSO; ^#^ *p* < 0.05, ^##^ *p* < 0.01 vs. NT.

Treatment of Met1 (Figure 11) and IC8 (Figure 12) cells with increasing concentrations of BYL719 and GSK2636771 for 1 h confirmed a dose-dependent reduction of Akt phosphorylation upon p110α but not p110β inhibition in these cells. Similar results were obtained upon incubation with the inhibitors for 24 h (Supplementary Figures S9 and S10), although, in these conditions, treatment with 1 µM GSK2636771 also appeared to affect pSer473 Akt slightly in Met1 cells (Supplementary Figure S9). Reduced Akt phosphorylation was also observed upon treatment with another p110α inhibitor, A66, but not with the p110γ inhibitor AS252424 in IC8 (Supplementary Figure S11a) and Met1 (Supplementary Figure S11b) cells. Preliminary experiments further suggested that combination of BYL719 and GSK2636771 did not increase or it only slightly increased the effect of BYL719 in IC8 and Met1 cells respectively (Supplementary Figure S12).

Taken together these data demonstrate that p110α is the main isoform regulating Akt activation in IC8 and Met1 cells, consistent with data on cell growth and viability.

**Figure 13:**
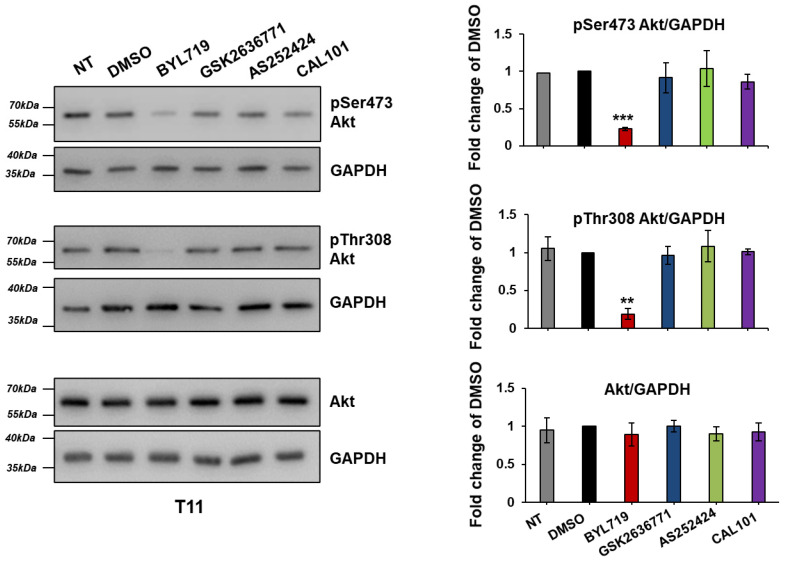
T11 cells were left untreated (NT) or treated with 1 µM of the isoform-specific inhibitors or DMSO in complete medium supplemented with 10% FBS. After 1 h, cells were Lysed and lysates were analyzed Western blotting. GAPDH was used as loading control. Signals were visualized using Chemidoc™ MP Imaging System. Graphs indicate results from densitometry analysis of phosphorylated or total Akt normalized to loading control and expressed as fold change of results from DMSO-treated cells. Data are means ± SEM from the following numbers of independent experiments: pSer473 Akt, n = 3 (apart from NT, n = 1 and BYL719, n = 2); pThr308 Akt and total Akt, n = 3 (apart from NT, n = 2). ** *p* < 0.01, *** *p* < 0.001 vs. DMSO.

We next investigated the effect of the isoform-specific inhibitors on T11 and IC1 cells. Data revealed that treatment with BYL719 for 1 h was able to inhibit Akt phosphorylation in T11 cells without affecting total levels of Akt (Figure 13, Supplementary Figure S13), similarly to results obtained in Met1 and IC8 cells. None of the other inhibitors affected Akt phosphorylation at the tested concentration (Figure 13, Supplementary Figure S13).

**Figure 14:**
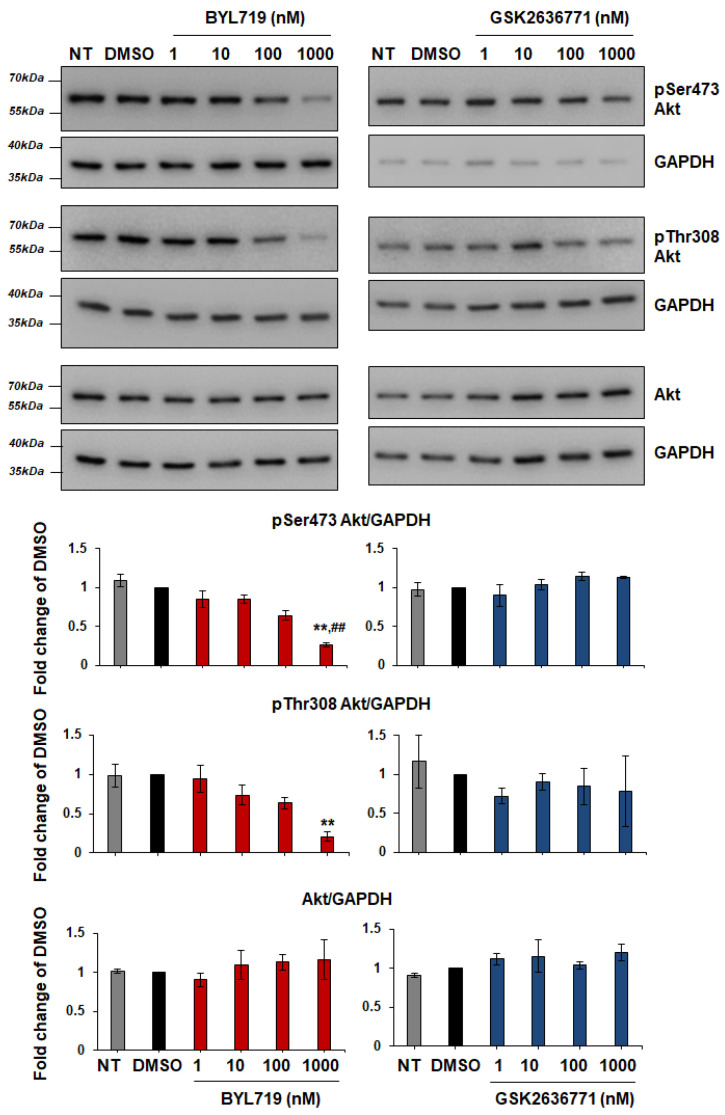
BYL719 inhibits Akt phosphorylation in T11 cells in a dose-dependent manner. T11 cells were treated for 1 h with increasing concentrations of BYL719 and GSK2636771 in complete medium supplemented with 10% FBS. Control cells were left untreated (NT) or treated with DMSO. Phosphorylation and total levels of Akt were analyzed by Western blotting, with GAPDH used as loading control. Signals were visualized using Chemidoc™ MP Imaging System. Graphs show results from densitometry analysis of phosphorylated or total Akt normalized to loading control and expressed as fold change of results from DMSO-treated cells. Data are means ± SEM from the following numbers of independent experiments: pSer473 Akt, n = 3 (apart from GSK2636771 1 µM, n = 2); pThr308 Akt, n = 3 (apart from NT in BYL719 graph and GSK2636771 1 µM, n = 2); Akt, n = 3 (apart from NT in BYL719 graph and GSK2636771 100 nM and 1 µM, n = 2); ** *p* < 0.01 vs. DMSO; ^##^ *p* < 0.01 vs. NT.

Dose response analysis confirmed inhibition of Akt phosphorylation in T11 cells treated with BYL719 but not with GSK2636771 (Figure 14).

Inhibition of Akt phosphorylation was also detected in IC1 cells upon 1 h (Supplementary Figure S14a) or 24 h (Supplementary Figure S14b) treatment with BYL719.

These data indicate that BYL719 inhibits Akt activation in all cSCC cell lines. Together with our previous data, these results indicate that the limited effect of BYL719 on IC1 and T11 cell growth and viability is not due to a generic lack of response to the drug in these cells.

## 3. Discussion

Data in the literature indicate that the PI3K/Akt/mTOR pathway could be a valid target in cSCC [26,38]. The potential beneficial effects of targeting mTOR is supported by preclinical studies [39,40,41,42] as well as evidence from organ transplant recipients [43,44,45,46,47], who have increased risk of cSCC because of prolonged immunosuppression [8]. Whether targeting selective PI3K isoforms can also prove beneficial remains to be fully established.

In this study we assessed the sensitivity to pan and isoform-specific PI3K inhibition of four cell lines, part of a recently described panel of patient-derived cSCC cell lines [34,35]. Importantly, published cSCC cell lines are few and this panel is among the largest [34,35]. The effect of the inhibitors on growth and viability of immortalized keratinocyte cell lines was also investigated. We show that simultaneous inhibition of all PI3K isoforms or blockade of mTOR reduced growth and viability of all investigated cell lines. As the tested cSCC cells derive from either moderately or poorly differentiated primary tumors [34], these results are consistent with data from our previous mutational pathway analysis of 40 primary cSCC tumors that identified the PI3K/Akt pathway amongst the KEGG signaling pathways that were more mutated in the moderately differentiated/poorly differentiated group [35].

Using isoform-specific inhibitors we further show that the p110α inhibitor BYL719 inhibited growth and viability of the cSCC cell lines IC8 and Met1 more potently than any of the other inhibitors. Similar results were obtained in immortalized keratinocyte cell lines. On the other hand, neither BYL719 nor any of the other selective inhibitors had any major or consistent effect on both cell counting and MTT assays in the cSCC cell lines IC1 and T11, when used at a concentration of 1 µM. These data indicate that, while growth and viability of all investigated cSCC cells is reduced by pan PI3Ks or mTOR inhibition, sensitivity to selective PI3K inhibitors, in particular to BYL719, differs between these cells. It remains to be established whether these inhibitors might affect growth and viability of IC1 and T11 cells if used at concentrations higher than 1 µM. In this respect, it must be noted that treatment with 1 µM of GSK2636771 [48] or AS252424 [49] was able to reduce the number of prostate cancer PC3 cells [48] and pancreatic cancer HPAF-II and Capan1 cells [49] respectively. Similarly, 1 µM CAL101 induced apoptosis in diffuse large B-cell lymphoma, follicular lymphoma and B-cell acute lymphoblastic leukemia cell lines [50]. These data suggest that the concentration of the inhibitors used in this study should be able to inhibit their target PI3K. On the other hand, as the IC_50_s of these inhibitors towards their selective targets are in the low nanomolar range and other PI3Ks might be affected by high concentrations of the compounds [50,51,52,53], the possibility still remains that 1 µM BYL719 might have blocked more than one isoform in these experiments. Although some results were confirmed by using lower concentrations of this drug, additional studies are now required to confirm whether the effect of BYL719 on Met1 and IC8 cells is due to sole inhibition of p110α. It must be noted that, although the majority of our results were consistent between the two assays, we detected statistically significant effects on cell counting but not on MTT assays in a few instances. As this occurred mostly when cell numbers were only slightly reduced, the apparent discrepancy might be due to a lower sensitivity of MTT assays in these experiments. On the other hand, the reason for observing different effects of CAL101 on counting and MTT assays in T11 cells (Figure 8c,d) would require further investigation. In addition, the p110γ inhibitor AS252424 appeared to have an effect in MTT but not in cell counting assays in IC8 cells (Figure 6a,b) which might be due to a specific effect of the inhibitor on mitochondrial activity rather than viability of these cells. The conclusions discussed in this study are based on results that were consistent between the two assays.

Overall, our data indicate that growth and viability of IC1 and T11 cells are more resistant to BYL719 treatment compared to IC8 and Met1 cells. The differential sensitivity to BYL719 was not due to different expression levels of p110α as this was fairly consistent between all cell lines used in this study. Furthermore, data in the literature indicate that activating mutations of *PIK3CA*, detected in many cancers [54], do not occur at high frequency in primary cSCC [55,56]. Interestingly, clinically-relevant genomic alterations of *PIK3CA* have been reported in some recurrent, metastatic cSCC [57] and *PIK3CA* activating mutations have been detected in some cSCC lymph node metastases [58]. These observations, together with results from a recent study reporting the inhibitory effect of the PI3K inhibitor PIK-75 on cell lines derived from cSCC metastases of the head and neck [59], strongly suggest that sensitivity to BYL719 should be further investigated in a panel of metastatic cSCC cells.

Resistance to p110α inhibition has been documented [60,61,62] and compensatory roles of other PI3Ks, in particular of p110β, have been described [36,37]. Our observation that sensitivity to BYL719 did not appear to depend on expression levels of p110β and that simultaneous inhibition of p110α and p110β did not affect numbers or viability of IC1 and T11 cells, however, rules out a compensatory role of p110β in the tested cSCC cells, at least in the experimental conditions used in this study. As cell numbers and viability of IC1 and T11 cells was strongly inhibited by the pan PI3K inhibitor LY294002 further investigation is required to establish whether other PI3Ks, including members of the class II subfamily, contribute to growth/viability of these cells in normal growing conditions or upon p110α inhibition. Overexpression of the receptor tyrosine kinase Axl has been involved in resistance to BYL719 in head and neck squamous cell carcinoma (HNSCC) and esophageal squamous cell carcinoma (ESCC) [62]. Interestingly, Axl not only is expressed in Met1 and IC1 cells [63] but is also upregulated in Met1 cells compared to their premalignant cells [64] and in cSCC cells compared to primary normal human keratinocytes [35]. Moreover, depletion of Axl was shown to reduce resistance of cSCC cell lines to chemotherapy drugs [63]. Although our experiments were performed within a short time frame, it would be worth investigating whether an upregulation of Axl might be involved in the reduced response to BYL719 of IC1 and T11 cells. In this respect, it must be noted that resistance to BYL719 mediated by Axl occurs through an Akt-independent mTOR activation in HNSCC and ESCC [62]: this would be consistent with our observation that growth and viability of both IC1 and T11 cells are reduced by mTOR inhibition. Recently, it has been reported that combination of BYL719 with a c-Jun N-terminal kinase inhibitor induced Axl downregulation and mTOR inhibition, resulting in a synergistic anti-proliferative effect in vitro [65]. It would be interesting to determine whether such a combination might prove effective on IC1 and T11 cells.

While BYL719 did not affect their viability and growth, it was able to inhibit Akt activation in IC1 and T11 cells in the experimental conditions used in this study. Whether a rebound Akt activation occurs in these cells upon longer incubation with the inhibitor remains to be established. Nonetheless, these data support the conclusion that monitoring Akt phosphorylation might not be directly indicative of the efficacy of the inhibitor on viability and growth of cSCC cells, as previously observed in breast cancers [36]. It must also be noted that phosphorylation of Akt in this study was assessed by using antibodies that do not discriminate between Akt1 and Akt2. As the two isoforms play distinct roles in epidermis [66] and are known to be regulated differently during cSCC progression [29], it would be important to monitor the specific effect of BYL719 on each Akt isoform.

Finally, as it was limited to four cSCC cell lines, our study cannot provide a final answer to the question of whether targeting specific PI3K(s) might represent a therapeutic strategy in cSCC, especially considering the many cross talks between this pathway and other signaling cascades. Important information can be obtained from our data nonetheless. Our results have revealed a heterogeneous response of cSCC cell lines to selective PI3K inhibitors (in particular p110α inhibition), possibly suggesting that sensitivity to these drugs might be patient-specific. The observation that BYL719 affects growth and viability of two of the investigated cSCC as well as two immortalized keratinocyte cell lines, however, might already suggest some limitations in the potential use of this inhibitor. Clearly, more studies are now needed to validate whether selective PI3K inhibitors have a therapeutic value in cSCC. Some initial data from this study were presented, as a poster, at the British Society for Investigative Dermatology Annual Meeting Norcroft Conference Centre, University of Bradford, Bradford, UK, 1–3 April 2019 [67].

## 4. Materials and Methods

### 4.1. Cell Culture and Treatment

cSCC cell lines, previously characterized [34,35], and immortalized keratinocytes N-TERT and Neb1 cells were cultured in Dulbecco’s Modified Eagle Medium/Nutrient Mixture F-12 (DMEM/F-12 GlutaMAX™ supplement, Gibco™, Thermo Fisher Scientific, Waltham, MA, USA, cat no: 10565018). Immortalized keratinocytes HaCaT cells were cultured in DMEM (Gibco™, Thermo Fisher Scientific, Waltham, MA, USA, cat no: 12077549). Media were supplemented with 10% Fetal Bovine Serum (FBS) (Gibco™, Thermo Fisher Scientific, Waltham, MA, USA, cat no: 11550356) and 1% Penicillin-Streptomycin (Gibco™, Thermo Fisher Scientific, Waltham, MA, USA, cat no: 11548876). Cells were incubated at 37 °C, 5% CO_2_. The following inhibitors were used in this study: BYL719 (Cambridge Bioscience, Cambridge, UK, cat no: CAY16986), GSK2636771 (Generon, Slough, UK, cat no: B2186), AS252424 (Generon, Slough, UK, cat no: SY-AS-252424), CAL101 (Cambridge Bioscience, Cambridge, UK, cat no: c0044), LY294002 (Cambridge Bioscience, Cambridge, UK, cat no: CAY70920), rapamycin (Cambridge Bioscience, Cambridge, UK, cat no: CAY13346), A66 (Tocris Bioscience, Bristol, UK, cat no: 5595). Cells were incubated with each inhibitor at a concentration of 1 µM (unless otherwise stated) in complete medium supplemented with 10% FBS.

### 4.2. Cell Counting and MTT Assays

Cells were seeded in 12 well plates (cell counting) or 48 wells (MTT assays) and treated with the indicated inhibitors 24 h after plating. Control cells were treated with vehicle alone (DMSO). After 72 h, cells were manually counted using a Burker chamber and a light microscope at 10x magnification. All experiments were performed in duplicate. Alternatively, cells were incubated with 500 µg/mL Thiazolyl Blue Tetrazolium Bromide (MTT, Sigma Aldrich, St. Louis, MO, USA, cat no: M2128) during the last 3 h of incubation. MTT formazan crystals were then dissolved with DMSO and absorbance at 570 and 650 nM was measured using an absorbance microplate reader.

### 4.3. Western Blotting Analysis

Cells were seeded in 6 well plates. For analysis of PI3Ks expression levels, cells in normal growing conditions were used. Alternatively, cells were treated with inhibitors for 1 h or 24 h before lysis. For experiments presented in Figure 11, Figure 12 and Figure 14, two 6 well plates were plated in parallel and used for the BYL719 and GSK2636771 dose response experiments respectively. In each plate, one well was left untreated (NT) and one was treated with vehicle (DMSO) alone as controls. Cells were lysed using 2% SDS, followed by sonication. Protein concentration was assessed using Pierce™ BCA Protein Assay Kit (Thermo Fisher Scientific, Thermo Scientific™, Waltham, MA, USA, cat no: 23227). Samples were separated by SDS-PAGE and transferred into nitrocellulose membranes. Membranes were incubated with 5% skimmed milk in Phosphate-buffered saline supplemented with 0.05% (*v*/*v*) Tween 20 (PBS-T) for 30 min at room temperature, followed by overnight incubation with primary antibodies at + 4 °C. Primary antibodies (and the corresponding dilutions, in PBS-T) were as follow: anti p110α (Cell Signaling Technology, Danvers, MA, USA, cat no: 4249, 1:1000), anti p110β (Cell Signaling Technology, Danvers, MA, USA, cat no: 3011, 1:1000), anti p110γ (Cell Signaling Technology, Danvers, MA, USA, cat no: 4252, 1:1000), anti p110δ (Santa Cruz Biotechnology, Dallas, TX, USA, cat no: SC-55589, 1:1000), anti pSer473 Akt (Cell Signaling Technology, Danvers, MA, USA, cat no: 4060, 1:1000), anti pThr308 Akt (Cell Signaling Technology, Danvers, MA, USA, cat no: 2965, 1:1000), anti Akt (Cell Signaling Technology, Danvers, MA, USA, cat no: 4691, 1:1000), anti α-Tubulin (Sigma Aldrich, St. Louis, MO, USA, cat no: T9026, 1:20,000); anti Vinculin (Sigma Aldrich, St. Louis, MO, USA, cat no: V9131, 1:10,000); anti GAPDH (Cell Signaling Technology, Danvers, MA, USA, cat no: 5174, 1:5000). After washing with PBS-T, membranes were incubated with peroxidase conjugated secondary antibodies (anti-rabbit IgG, cat no: A6154, 1:10,000; anti-mouse IgG, cat no: A0168, 1:10,000, both from Sigma Aldrich, St. Louis, MO, USA) for 1 h at room temperature, washed with PBS-T and exposed to ECL reagent (Sigma Aldrich, St. Louis, MO, USA, Immobilon™ Western Chemiluminescent HRP Substrate, cat no: WBKLS0500). Signals were visualized using X-ray Film 18 cm × 24 cm Double Sided (Scientific Laboratory Supplies, Nottingham, UK cat no: MOL7016) using a film processor or using Chemidoc™ MP Imaging System (Bio-Rad, Hercules, CA, USA). For experiments visualized using X-ray films and a film processor, membranes were cut into strips around the molecular weight of each protein of interest and incubated with the corresponding primary antibodies. For experiments visualized using Chemidoc™ Imaging System, the whole membranes were incubated with the specific primary antibody first. After visualization, membranes were re-incubated with anti GAPDH or anti Tubulin as loading control.

## 5. Conclusions

Although limited to four cSCC cell lines, our study has revealed their differential sensitivity to selective PI3K inhibition, confirming the highly heterogeneous nature of cSCC. This highlights the critical need for reliable experimental tools that can allow careful preclinical studies. In this respect, this study has contributed further to the characterization of a panel of cSCC cell lines, which will prove very useful in preclinical testing to identify novel therapeutic strategies.

## Supplementary Materials

The following are available online at https://www.mdpi.com/article/10.3390/ijms22073567/s1, Figure S1: Analysis of p110α and p110β expression in immortalized keratinocytes and cSCC cells and validation of loading control. Figure S2: Expression of p110γ and p110δ in cSCC cells. Figure S3: The PI3K/Akt/mTOR pathway regulates growth and viability of cSCC cell lines. Figure S4: Vehicle control does not affect number and viability of cells. Figure S5: BYL719 reduces numbers and viability of keratinocytes more potently than GSK2636771. Figure S6: BYL719 more potently than GSK2636771 reduces numbers and viability of IC8 and Met1 but not IC1 cells. Figure S7: BYL719 inhibits Akt phosphorylation in Met1 cells. Figure S8: BYL719 inhibits Akt activation in IC8 cells. Figure S9: BYL719 inhibits Akt activation in Met1 cells. Figure S10: BYL719 but not GSK2636771 inhibits Akt activation in IC8 cells. Figure S11: A66 inhibits Akt activation in IC8 and Met1 cells. Figure S12: BYL719 inhibits Akt activation in IC8 and Met1 cells. Figure S13: BYL719 inhibits Akt activation in T11 cells. Figure S14: BYL719 inhibits Akt activation in IC1 cells.

## Abbreviations

CNI	Calcineurin inhibitors
cSCC	Cutaneous squamous cell carcinoma
DMEM	Dulbecco’s Modified Eagle Medium
ESCC	Esophageal squamous cell carcinoma
FBS	Fetal Bovine Serum
HNSCC	Head and neck squamous cell carcinoma
ICI	Immune checkpoints inhibitors
KCs	Keratinocyte carcinomas
mTOR	Mechanistic (or mammalian) target of rapamycin
MTT	Thiazolyl Blue Tetrazolium Blue (MTT)
PBS-T	Phosphate-buffered saline supplemented with 0.05% Tween 20
PI3K	Phosphoinositide 3-kinase
PTEN	Phosphate and tensin homolog
PtdIns(3,4,5)*P_3_*	Phosphatidylinositol 3,4,5-trisphosphate
