# Supplementary material for: Growth and Viability of Cutaneous Squamous Cell Carcinoma Cell Lines Display Different Sensitivities to Isoform-Specific Phosphoinositide 3-Kinase Inhibitors
Source: Int J Mol Sci. 2021 Mar 30;22(7):3567. doi: 10.3390/ijms22073567 (PMC8036316; doi:10.3390/ijms22073567)
Supplement: Supplementary file 1 [file ijms-22-03567-s001.pdf]

Growth and viability of cutaneous squamous cell carcinoma cell lines display different sensitivities to isoform-specific phosphoinositide 3-kinase inhibitors

Viviana Mannella[1], Kira Boehm[1], Suheyla Celik[1], Tasnim Ali[1], Amnah N. Mirza[1], Mariam El Hasnaouy[1], Andreas Kaffa[1], Yanshuang Lyu[1,2], Donya Kafaei Golahmadi[1], Irene M. Leigh[3], Daniele Bergamaschi[1], Catherine A. Harwood[1] and Tania Maffucci[1,*]

Supplementary Material

Supplementary Figure 1

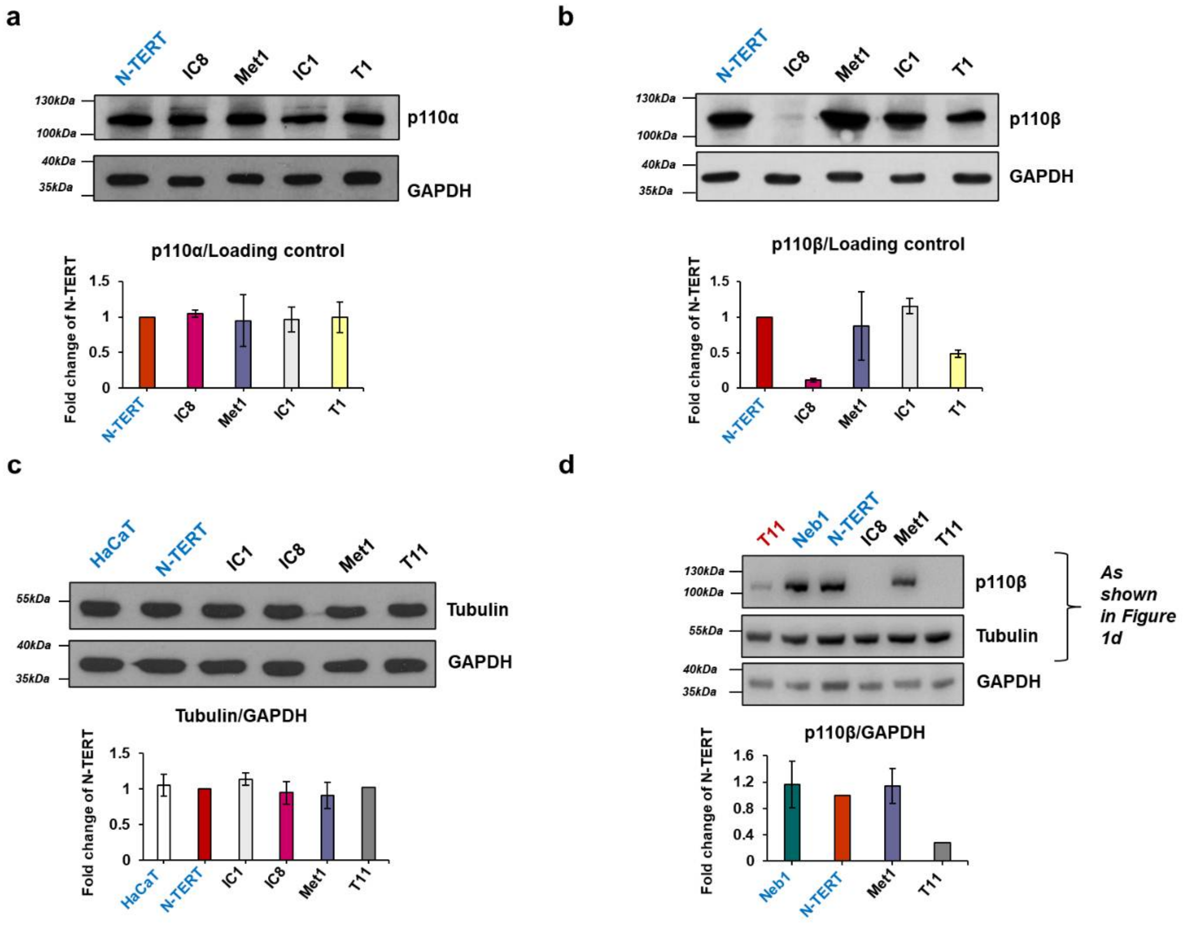

Supplementary Figure 1. Analysis of p110*α* and p110β expression in immortalized keratinocytes and cSCC cells and validation of loading control. Lysates from the indicated cell lines were separated by SDS/PAGE and analyzed by Western blotting using an anti p110*α* (**a**) or anti p110β (**b**,**d**) antibody. GAPDH or Tubulin were used as loading control. Alternatively, expression levels of Tubulin and GAPDH was assessed in lysates from the indicated cell lines (**c**). Signals were visualized using X-ray films and a film processor (**a**-**c**) or using Chemidoc™ MP Imaging System (**d**). Graphs represent results from densitometry analysis of the indicated proteins normalized to loading control levels and are expressed as fold change of results from N-TERT cells. Data in (**a**-**c**) are means ± SD from n = 2 sets of lysates prepared independently from separate batches of cells, apart from Met1 ((**a**), n = 9; (**b**), n = 7) and from T11 ((**c**), n = 1). Data in (**d**) are means ± SEM from n = 3 sets of lysates (apart from T11, n = 1). Note that blots in (**d**) are the same presented in Figure 1d, with the addition of the corresponding GAPDH.

Supplementary Figure 2

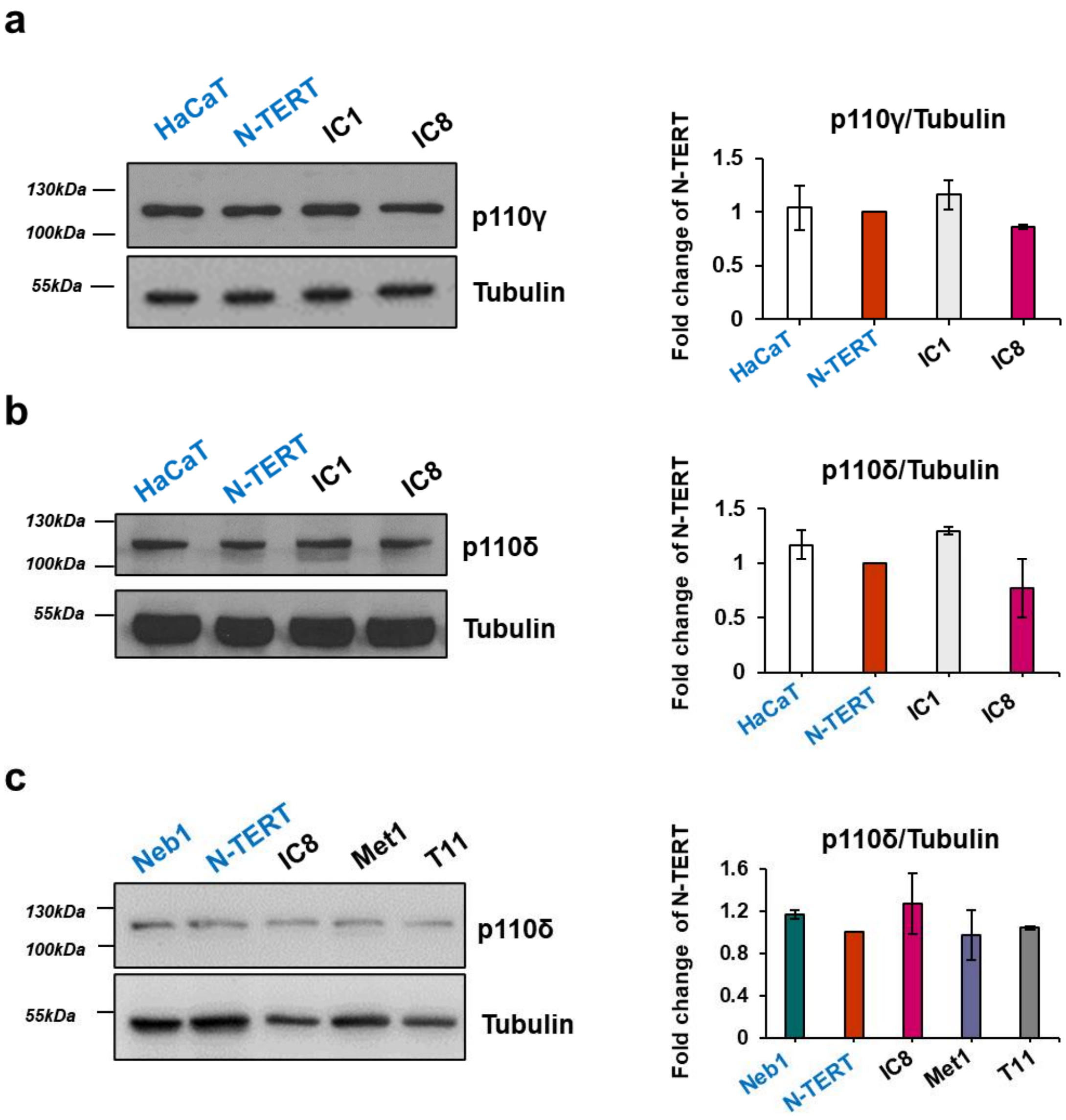

Supplementary Figure 2. Expression of p110γ and p110δ in cSCC cells. Lysates from the indicated cSCC cell lines were analyzed by Western blotting using the indicated primary antibodies. Tubulin was used as loading control. Signals were visualized using X-ray films and a film processor (**a**,**b**) or using Chemidoc™ MP Imaging System (**d**). Graphs indicate data from densitometry analysis for each enzyme, normalized to Tubulin and expressed as fold change of results from N-TERT cells. Data are means ± SD from n = 2 (**a**,**b**) and n = 3 (**c**) sets of lysates prepared independently.

Supplementary Figure 3

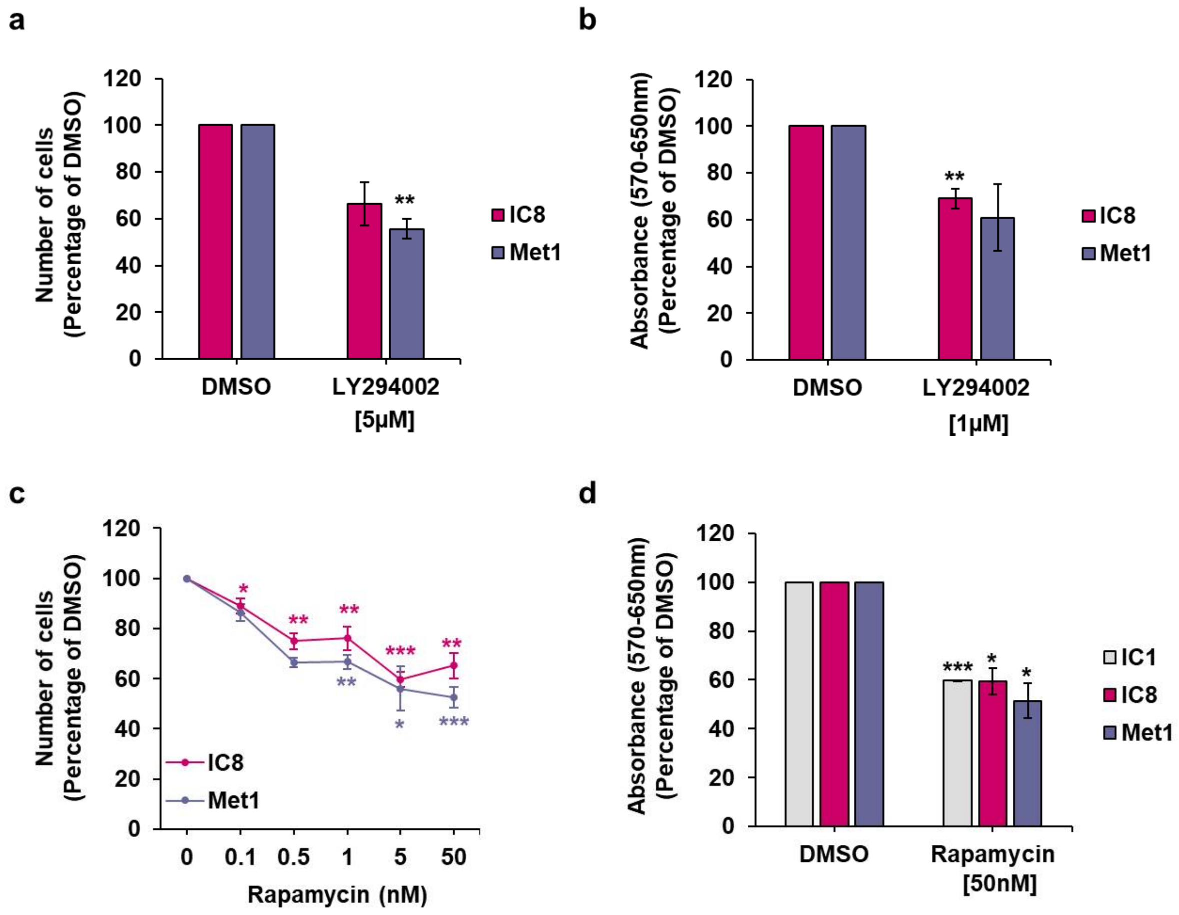

Supplementary Figure 3. The PI3K/Akt/mTOR pathway regulates growth and viability of cSCC cell lines. The indicated cSCC cell lines were treated with the indicated concentrations of LY294002 (**a**,**b**) or rapamycin (**c**,**d**) in complete medium supplemented with 10% FBS. Control cells were treated with vehicle alone (DMSO). After 72h, number of cells was assessed by cell counting (**a**,**c**) and cell viability was assessed by MTT assays (**b**,**d**). Data are expressed as percentage of results from control cells and are means ± SEM (apart from (**b**), means ± SD) from n = 3 (**a**), n = 3 ((**b**), IC8), n = 2 ((**b**), Met1), n = 4 ((**c**), IC8 apart from 1nM, n = 5), n = 3 ((**c**), Met1 apart from 0.5nM, n = 2 and 50nM, n = 5) and n = 3 (**d**) independent experiments performed in duplicate (**a**,**c**) or triplicate (**b**,**d**). * p <0.05, ** p <0.01, *** p <0.001 vs. DMSO.

Supplementary Figure 4

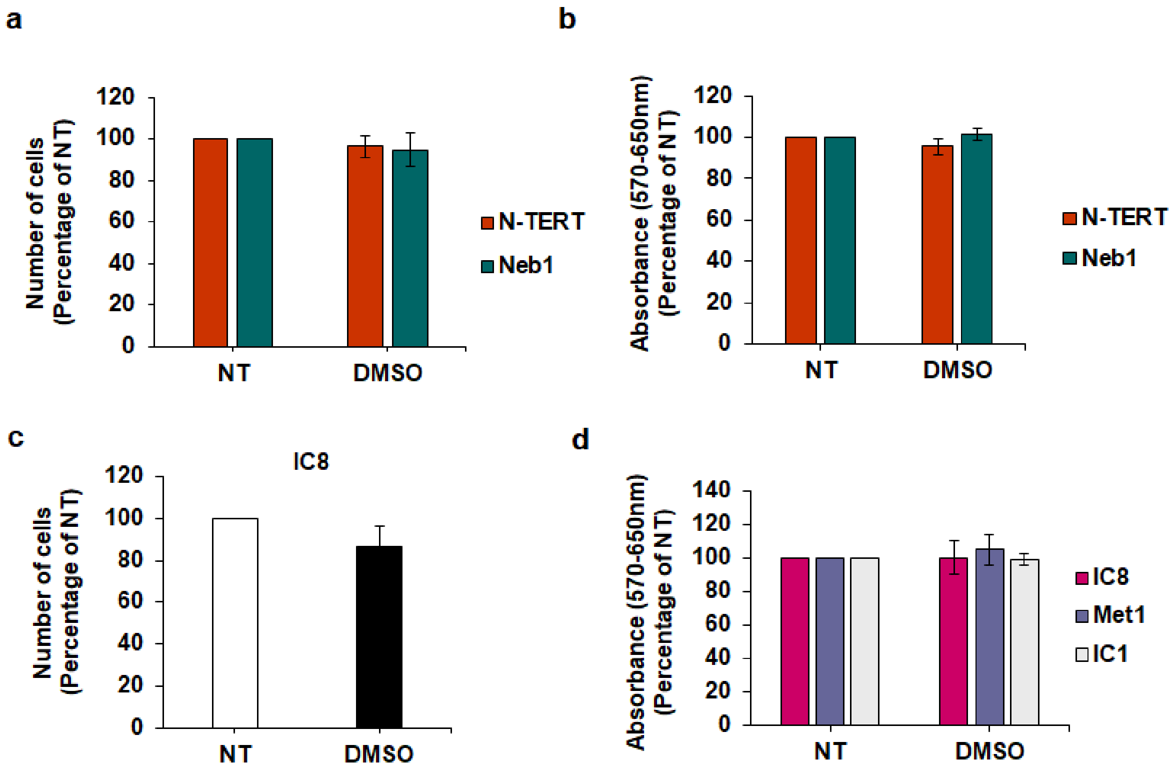

Supplementary Figure 4. Vehicle control does not affect number and viability of cells. N-TERT and Neb1 cells (**a**,**b**), IC8 (**c**,**d**), Met1 and IC1 (**d**) cells were left untreated (NT) or treated with vehicle (DMSO, 1:1,000) in complete medium supplemented with 10% FBS. After 72h, number of cells was assessed by cell counting (**a**,**c**) and cell viability was assessed by MTT assays (**b**,**d**). Data are expressed as percentage of results from untreated cells and are means ± SEM from n = 6 ((**a**), N-TERT), n = 4 ((**a**), Neb1), n = 13 ((**b**), N-TERT), n = 3 ((**b**), Neb1), n = 3 (**c**), n = 6 ((**d**), apart from Met1, n = 4) independent experiments performed in duplicate (**a**,**c**) or triplicate (**b**,**d**).

Supplementary Figure 5

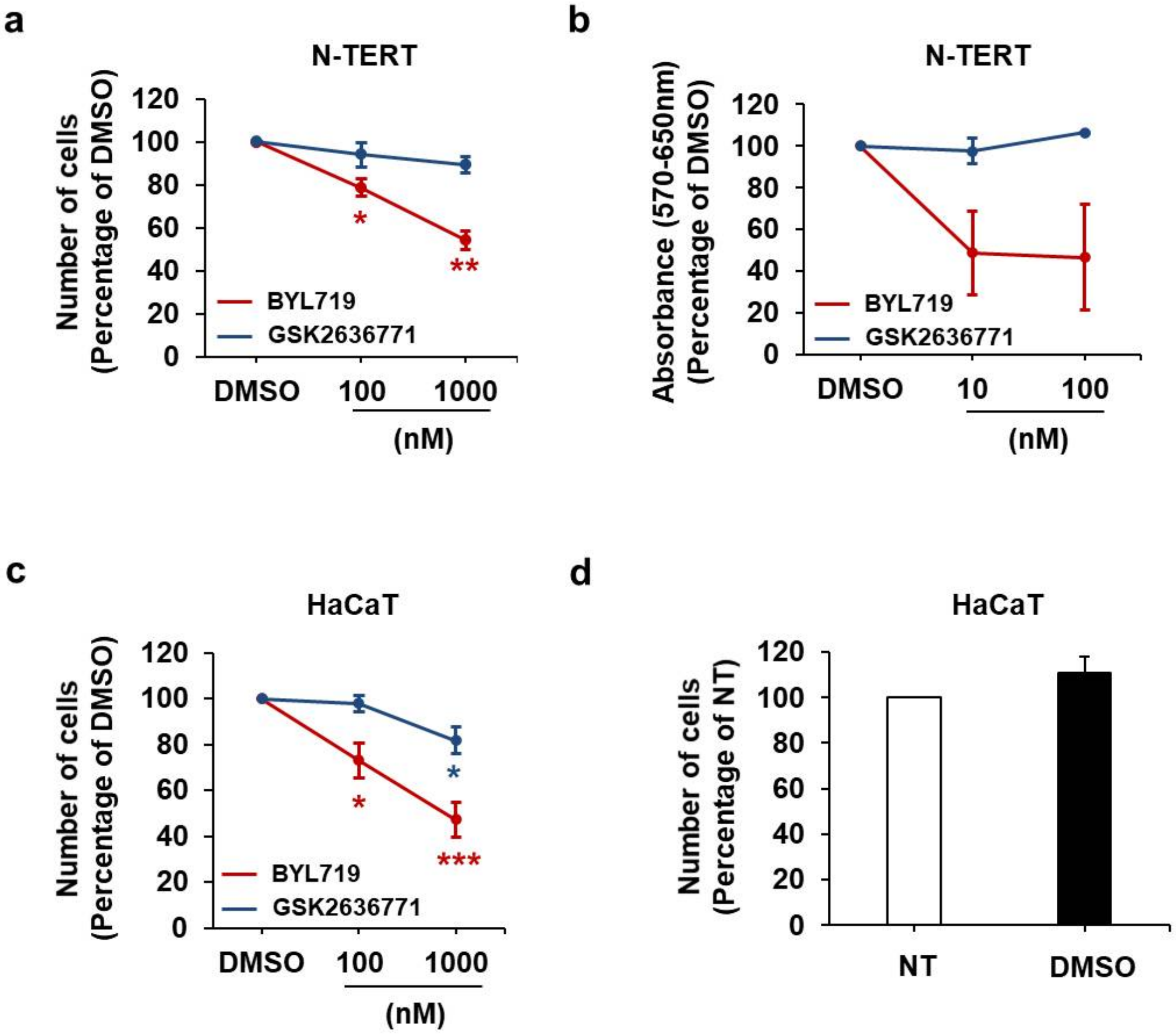

Supplementary Figure 5. BYL719 reduces numbers and viability of keratinocytes more potently than GSK2636771. N-TERT (**a**,**b**) and HaCaT (**c**) cells were treated with the indicated concentrations of BYL719 and GSK2636771 or vehicle (DMSO). Alternatively, HaCaT cells were left untreated (NT) or treated with DMSO (**d**). After 72h, number of cells was assessed by cell counting (**a**,**c**,**d**) and cell viability was assessed by MTT assays (**b**). Data are expressed as percentage of results from cells treated with DMSO (**a**-**c**) or untreated cells (**d**) and are means ± SEM (apart from (**b**), means ± SD) from n = 3 (**a**), n = 2 (**b**), n = 7 ((**c**), apart from GSK2636771 100nM, n = 4) and n = 4 (**d**) independent experiments performed in duplicate (**a**,**c**,**d**) or triplicate (**b**). * p <0.05, ** p <0.01, *** p <0.001 vs. DMSO.

Supplementary Figure 6

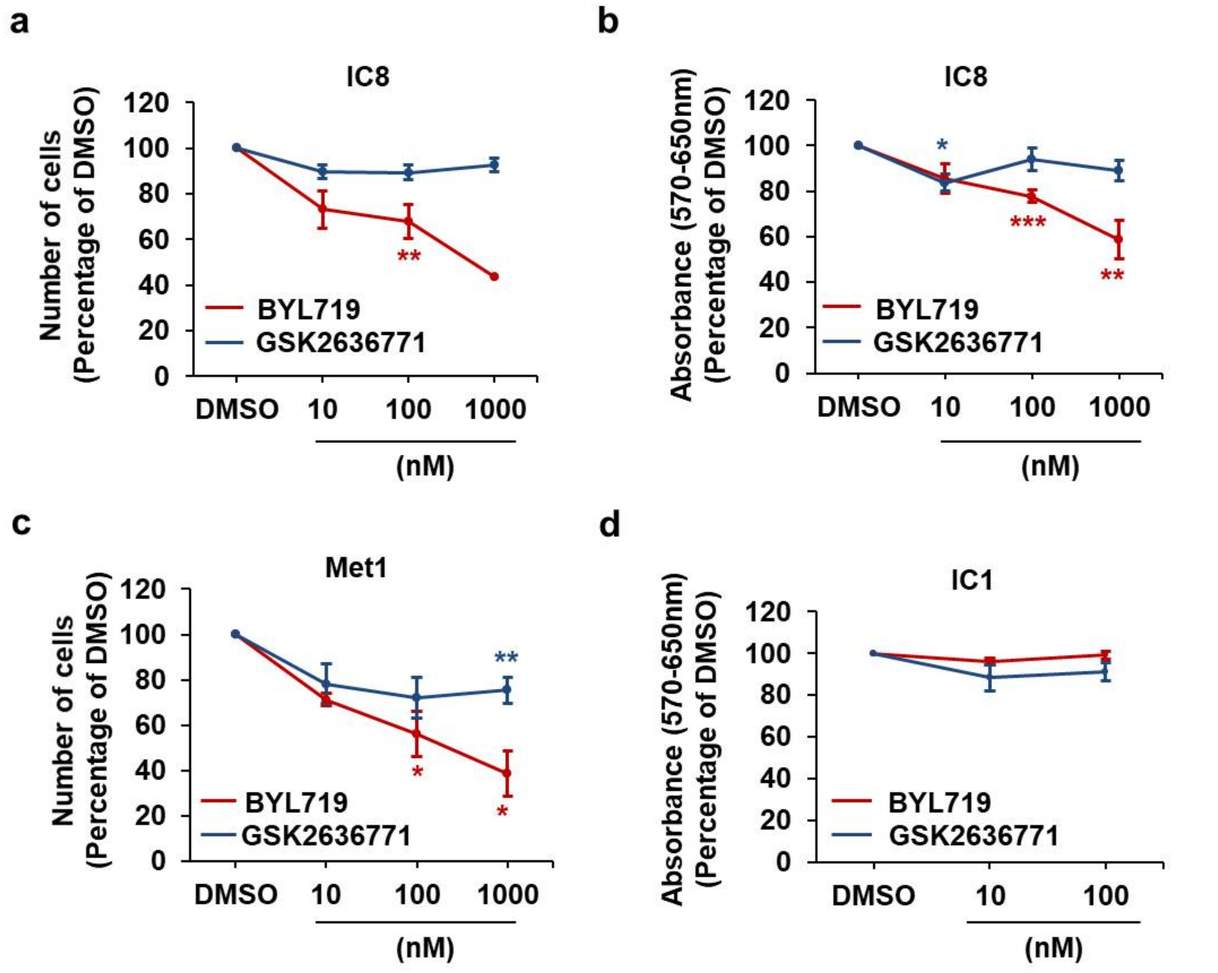

Supplementary Figure 6. BYL719 more potently than GSK2636771 reduces numbers and viability of IC8 and Met1 but not IC1 cells. IC8 (**a**,**b**), Met1 (**c**) and IC1 (**d**) cells were treated with the indicated concentrations of BYL719 and GSK2636771 or vehicle (DMSO). After 72h, number of cells was assessed by cell counting (**a**,**c**) and cell viability was assessed by MTT assays (**b**,**d**). Data are expressed as percentage of results from cells treated with DMSO and are means ± SEM from n = 3 ((**a**), apart from BYL719 100nM n = 6 , BYL719 1μM n = 1 and GSK2636771 1μM n = 4), n = 3 ((**b**), apart from BYL719 10 nM n = 4, BYL719 100nM n = 6 and BYL719 1μM n = 6), n = 4 ((**c**), apart from BYL719 10nM n = 2, BYL719 100nM, n = 5, BYL719 1μM n = 3, GSK2636771 1μM n = 9), and n = 8 ((**d**), apart from BYL719 100nM n = 12) independent experiments performed in duplicate (**a**,**c**) or triplicate (**b**,**d**). * $p < 0.05$, ** $p < 0.01$, *** $p < 0.001$ vs. DMSO.

Supplementary Figure 7

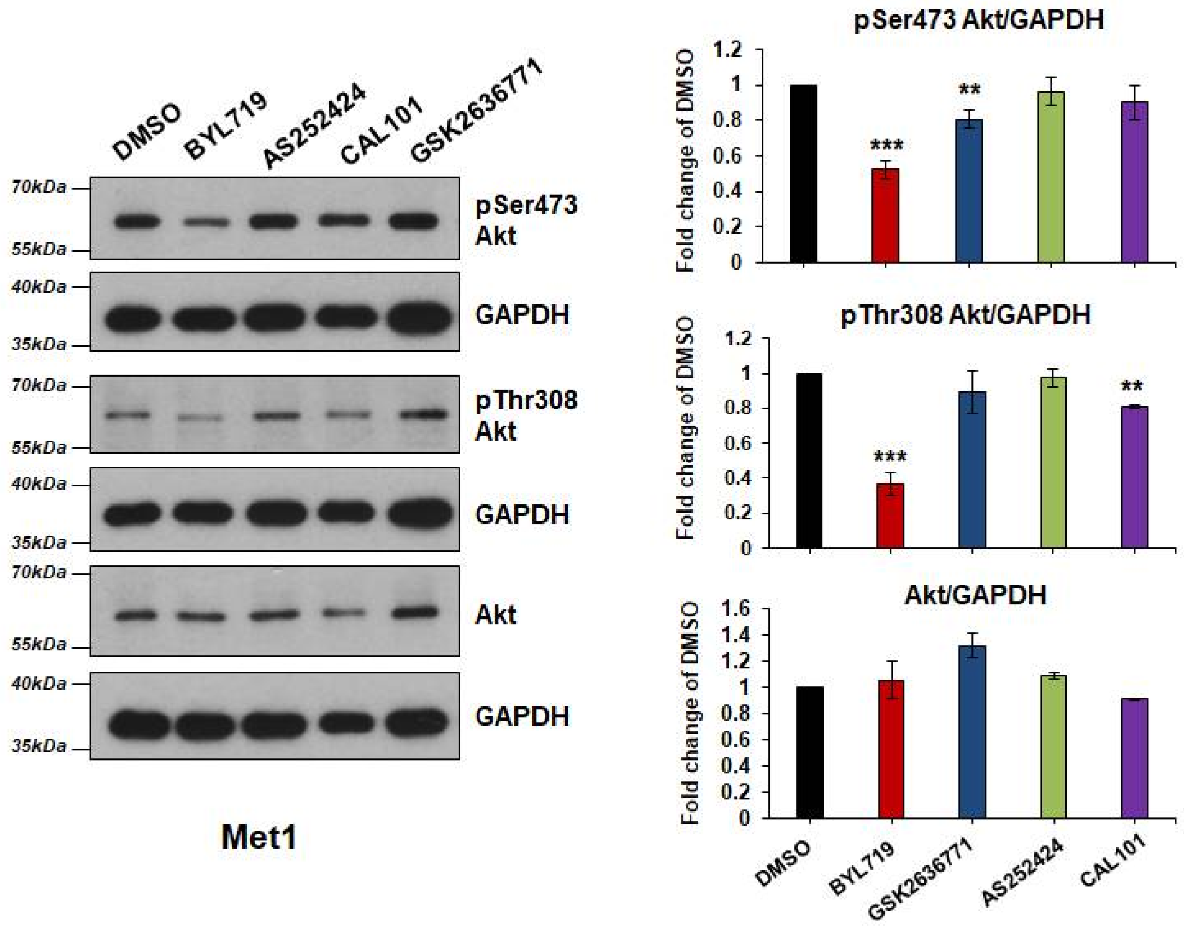

Supplementary Figure 7. BYL719 inhibits Akt phosphorylation in Met1 cells. Met1 cells were treated for 1h with 1μM of the isoform-specific inhibitors in complete medium supplemented with 10% FBS. Control cells were treated with DMSO. Akt phosphorylation status and total levels were determined by Western blotting. GAPDH was used as loading control. Signals were visualized using X-ray films and a film processor. Graphs indicate results from densitometry analysis of phosphorylated and total Akt normalized to loading control and expressed as fold change of results from DMSO-treated cells. Data are means ± SEM (apart from total Akt: means ± SD) from the following numbers of independent experiments: pSer473 Akt, n = 7-8; pThr308 Akt, n = 3-5; Akt, n = 2. ** p <0.01, *** p <0.001 vs. DMSO.

Supplementary Figure 8

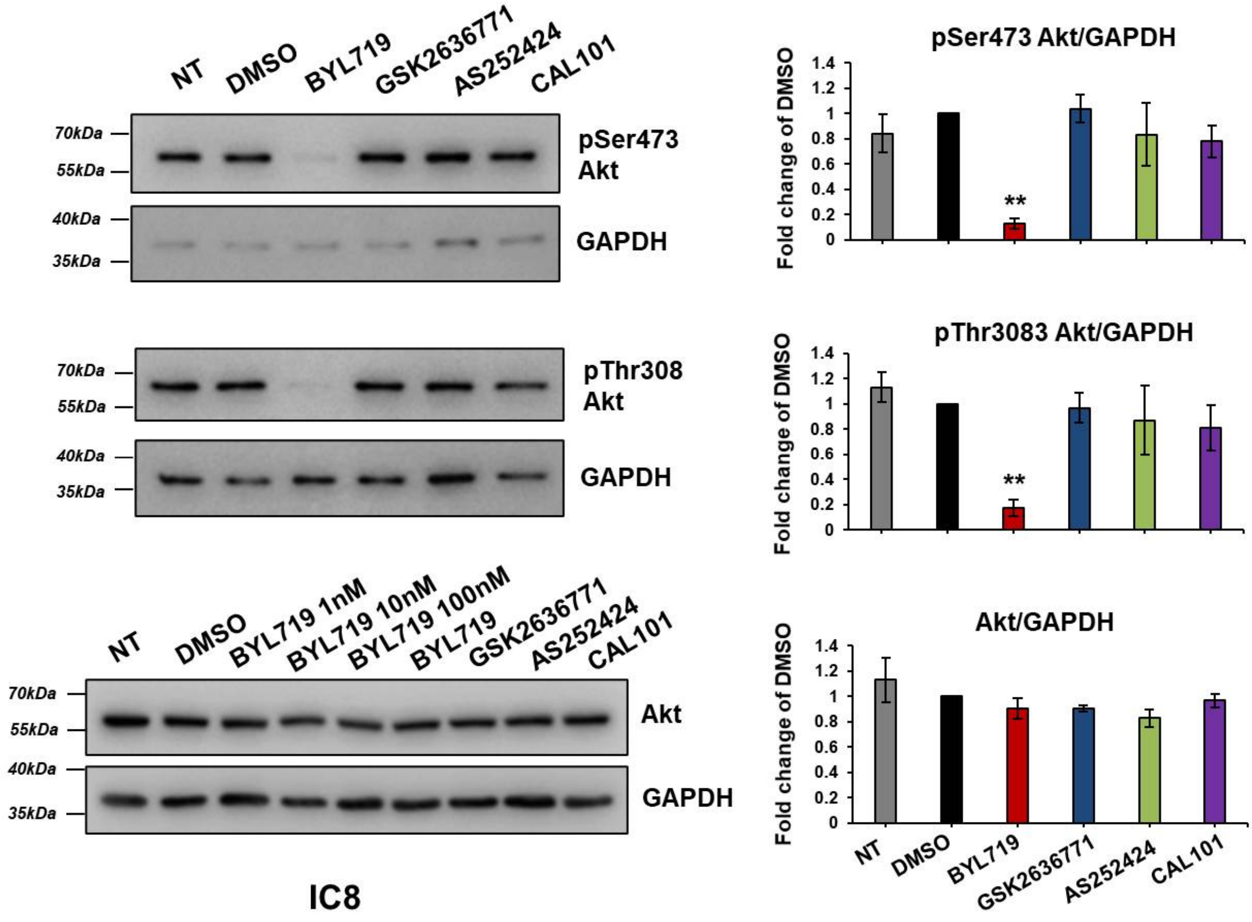

Supplementary Figure 8. BYL719 inhibits Akt activation in IC8 cells. IC8 cells were left untreated (NT) or treated with the indicated inhibitors or vehicle (DMSO) in complete medium supplemented with 10% FBS. Inhibitors were used at a final concentration of 1μM unless otherwise stated. After 1h, cells were lysed and analyzed by Western blotting. GAPDH was used as loading control. Signals were visualized using Chemidoc™ MP Imaging System. Graphs indicate results from densitometry analysis of phosphorylated and total Akt normalized to loading control and expressed as fold change of results from DMSO-treated cells. Data are means ± SEM from n = 3 independent experiments (apart from NT, n = 2). ** p <0.01 vs. DMSO.

Supplementary Figure 9

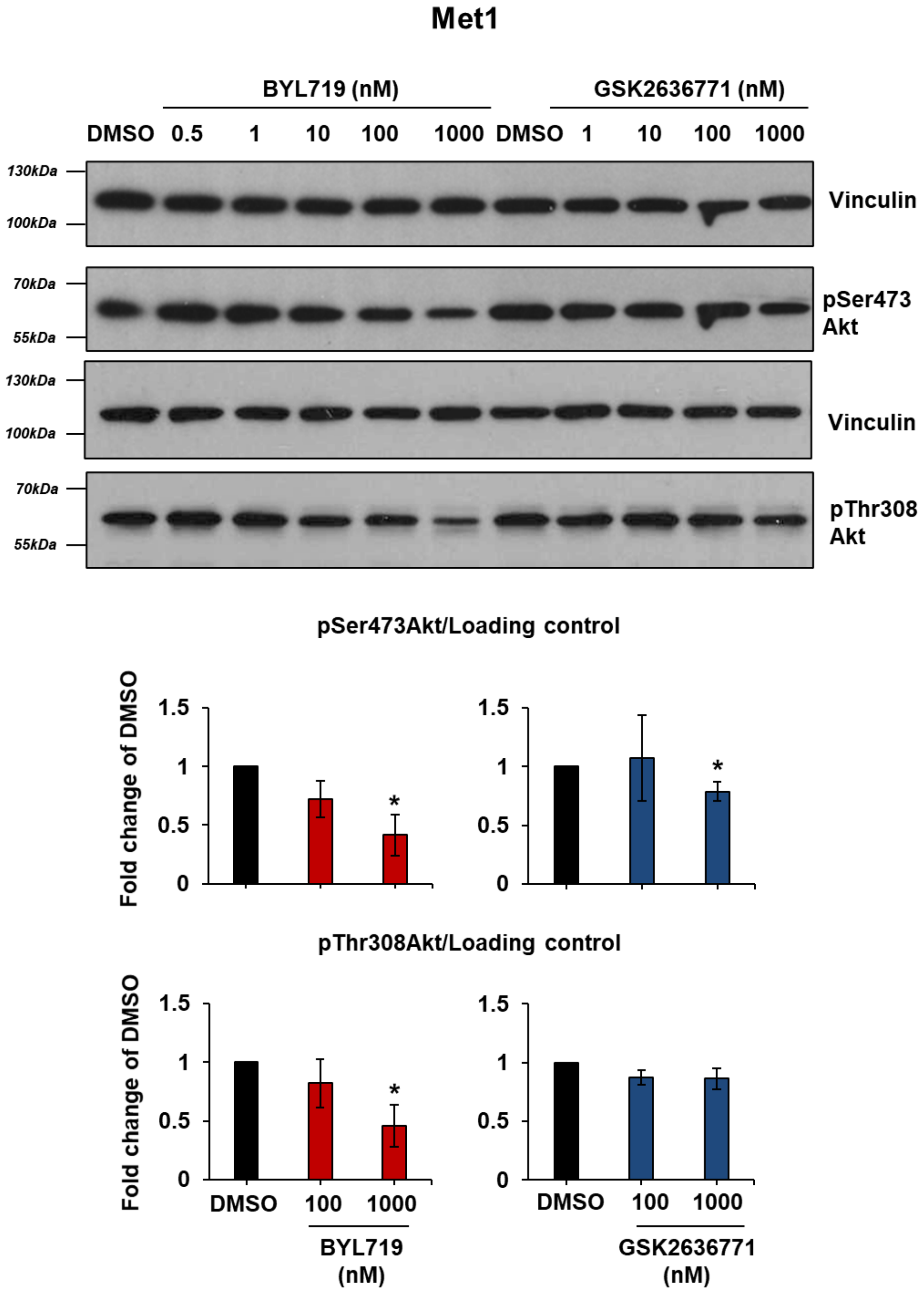

Supplementary Figure 9. BYL719 inhibits Akt activation in Met1 cells. Met1 cells were treated with increasing concentrations of the indicated inhibitors in complete medium supplemented with 10% FBS. Control cells were treated with DMSO. After 24h, cells were lysed and Akt phosphorylation at residues Ser473 and Thr308 was assessed by Western blotting. In these experiments, Vinculin or GAPDH were used as loading control. Signals were visualized using X-ray films and a film processor. Graphs indicate results from densitometry analysis of phosphorylated Akt normalized to loading control and expressed as fold change of results from control cells. Data are means ± SEM from n = 3-4 independent experiments (apart from GSK2636771 100nM, n = 2). * p <0.05 vs. DMSO (Student's t-Test, one-tailed distribution).

Supplementary Figure 10

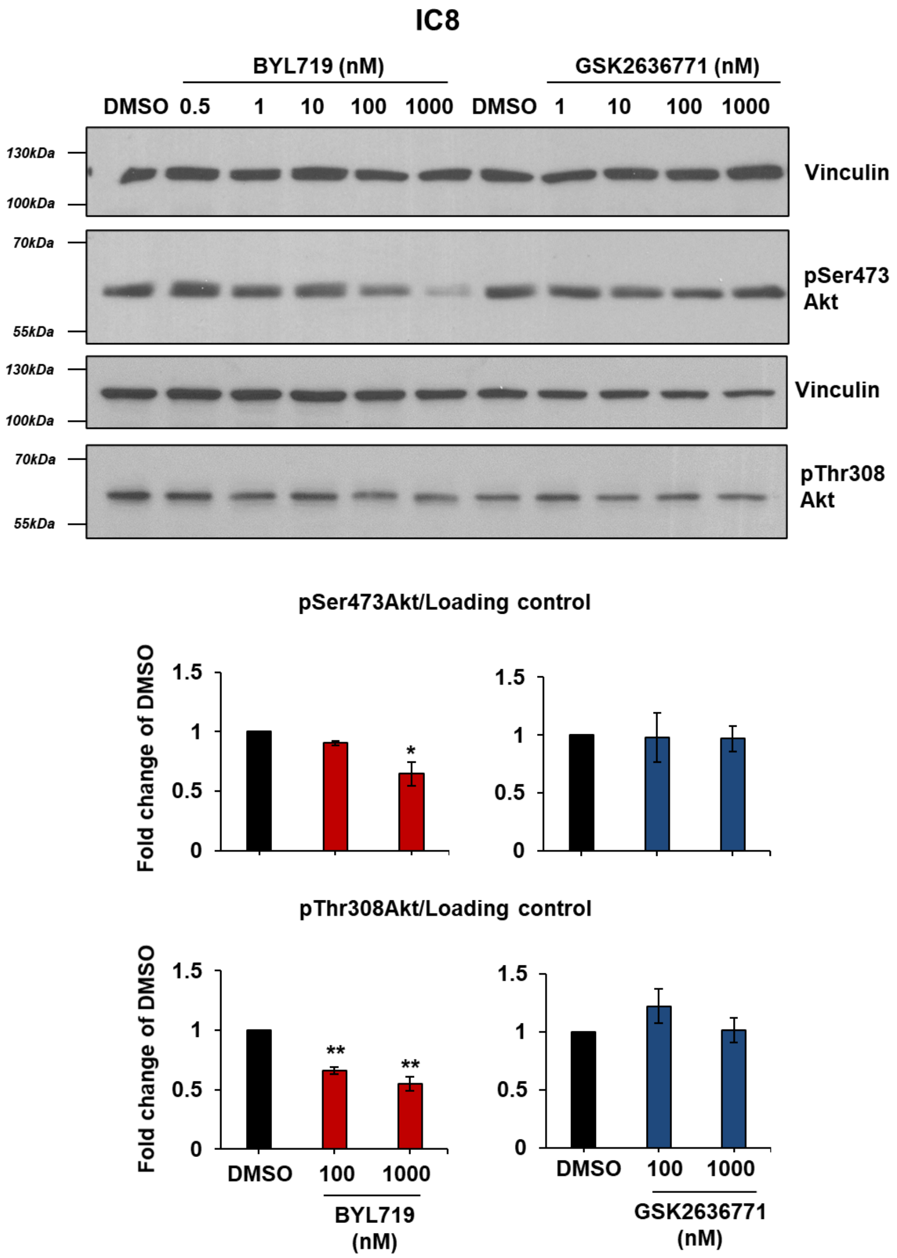

Supplementary Figure 10. BYL719 but not GSK2636771 inhibits Akt activation in IC8 cells. IC8 cells were treated for 24h with increasing concentrations of the indicated inhibitors or vehicle alone (DMSO) in complete medium supplemented with 10% FBS. Cells were lysed and Akt phosphorylation at residues Ser473 and Thr308 was assessed by Western blotting. In these experiments, Vinculin or GAPDH were used as loading control. Signals were visualized using X-ray films and a film processor. Graphs indicate results from densitometry analysis of phosphorylated Akt normalized to loading control and expressed as fold change of results from control cells. Data are means ± SEM from the following numbers of independent experiments: pSer473Akt, n = 3-5 (apart from BYL719 100nM, n = 2); pThr308Akt, n = 3-6. * p <0.05, ** p <0.01 vs. DMSO (Student's t-Test, one-tailed distribution).

Supplementary Figure 11

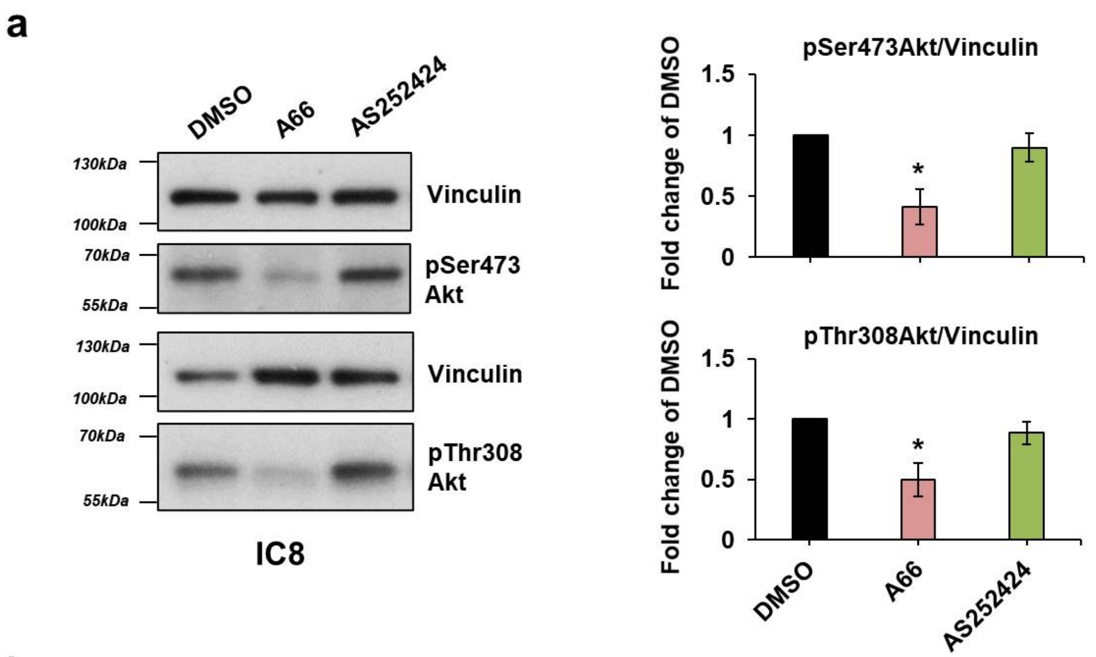

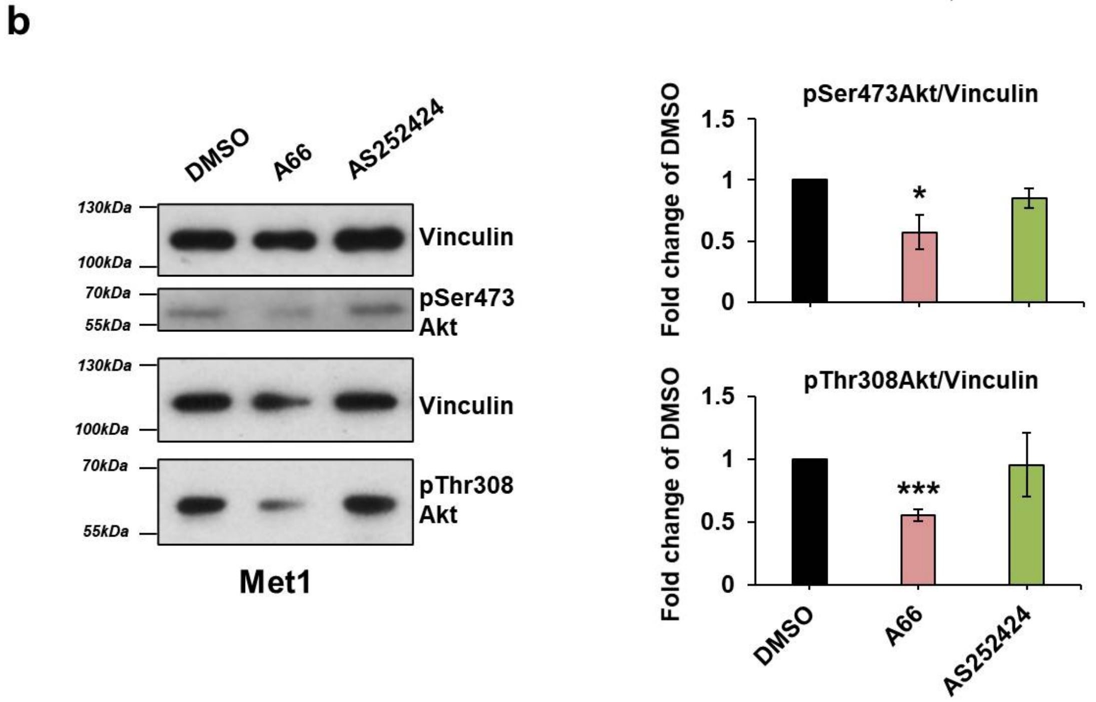

Supplementary Figure 11. A66 inhibits Akt activation in IC8 and Met1 cells. IC8 and Met1 cells were treated with the indicated inhibitors (1μM) in complete medium supplemented with 10% FBS. Control cells were treated with DMSO alone. After 24h, cells were lysed and analyzed by Western blotting using an anti pSer473 Akt or an anti pThr308 Akt antibody. Vinculin was used as loading control. Representative blots are shown. Graphs show results from densitometry analysis of phosphorylated Akt normalized to Vinculin levels. Data are expressed as fold change of results from control cells and are means ± SEM from n = 3-4 (**a**) and n = 5-6 (**b**) independent experiments. * p <0.05, *** p <0.001 vs. DMSO (Student's t-Test, one-tailed distribution).

Supplementary Figure 12

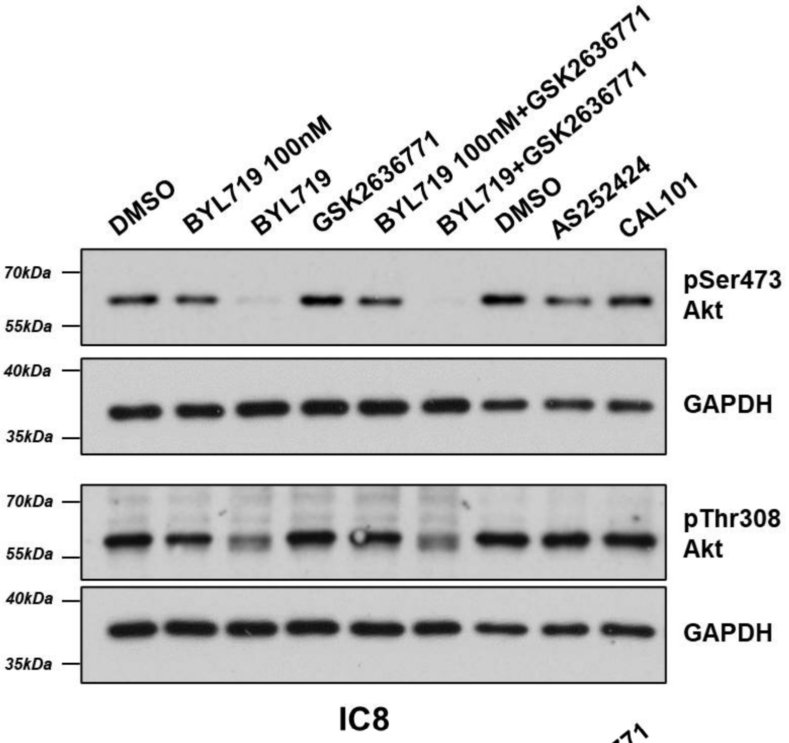

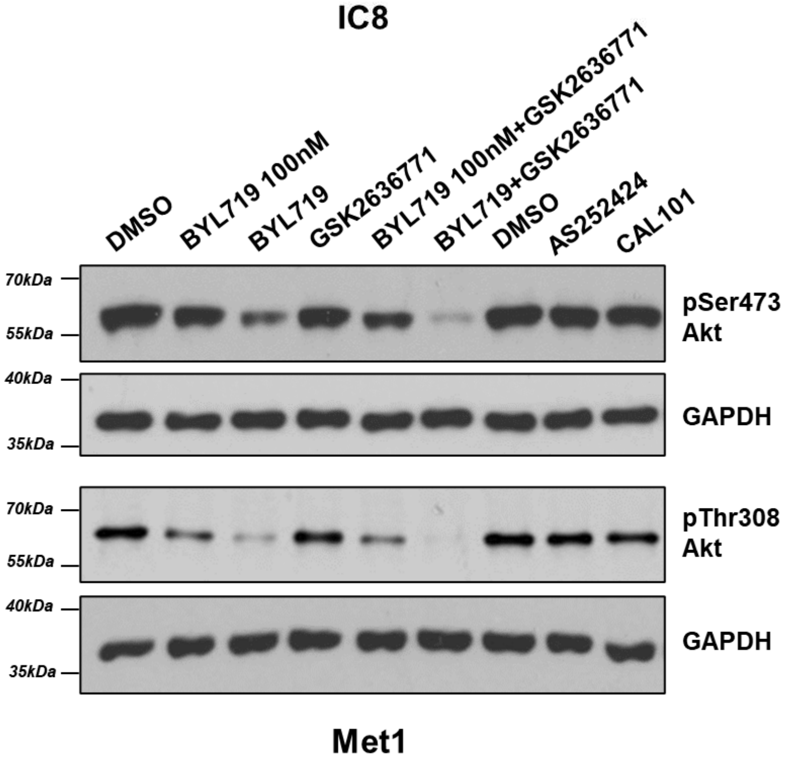

Supplementary Figure 12. BYL719 inhibits Akt activation in IC8 and Met1 cells. IC8 and Met1 cells were treated with the indicated inhibitors or vehicle (DMSO) in complete medium supplemented with 10% FBS. Inhibitors were used at a final concentration of 1μM unless otherwise stated. After 1h, cells were lysed and analyzed by Western blotting using an anti pSer473 Akt or an anti pThr308 Akt. GAPDH was used as loading control.

Supplementary Figure 13

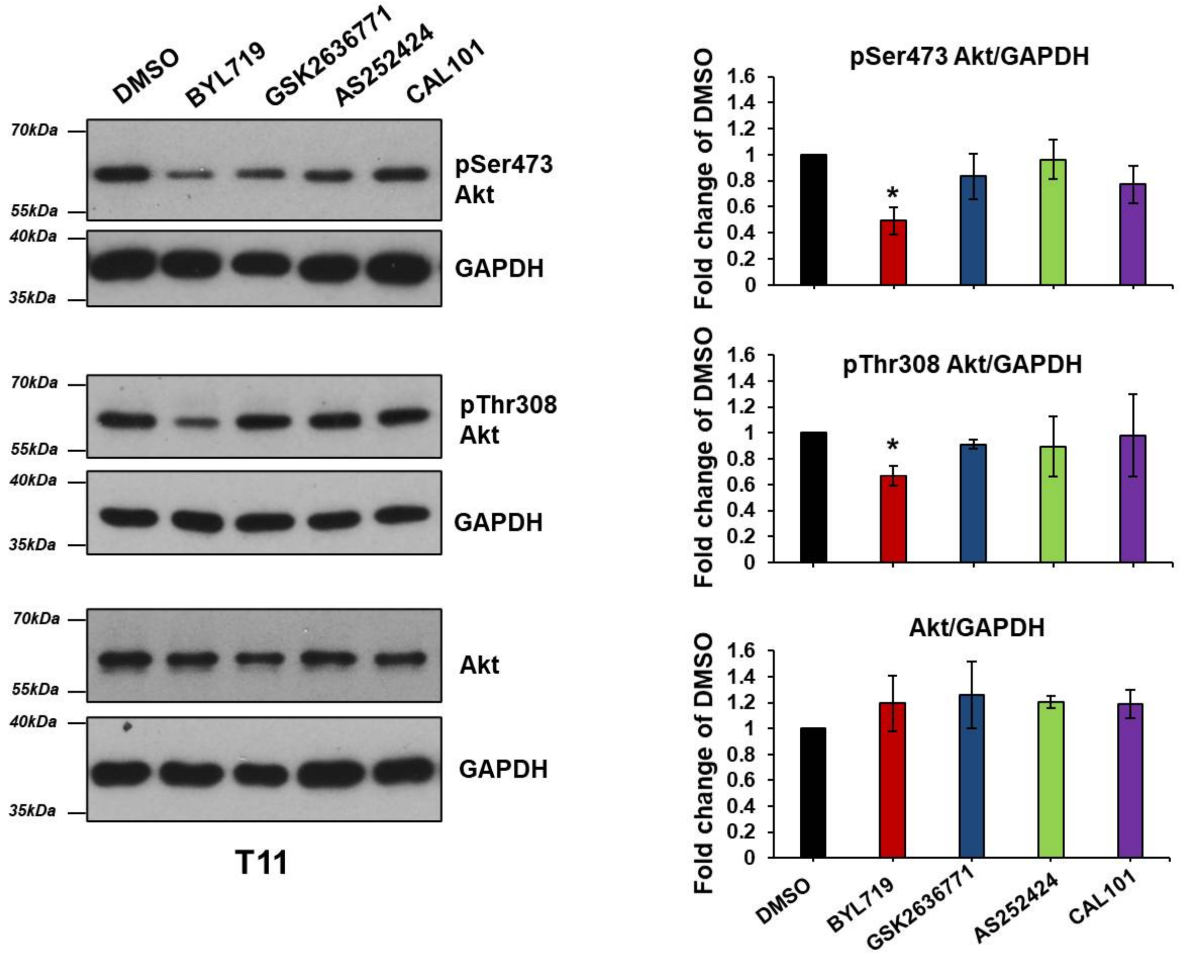

Supplementary Figure 13. BYL719 inhibits Akt activation in T11 cells. T11 cells were treated with 1μM of the isoform-specific inhibitors or DMSO in complete medium supplemented with 10% FBS. After 1h, cells were lysed and lysates were analyzed by Western blotting. GAPDH was used as loading control. Signals were visualized using X-ray films and a film processor. Graphs indicate results from densitometry analysis of phosphorylated or total Akt normalized to loading control and expressed as fold change of results from DMSO-treated cells. Data are means ± SEM from the following numbers of independent experiments: pSer473 Akt, n = 4; pThr308 Akt, n = 4 (apart from GSK2636771, n = 3) and total Akt, n = 3. * p <0.05 vs. DMSO.

Supplementary Figure 14

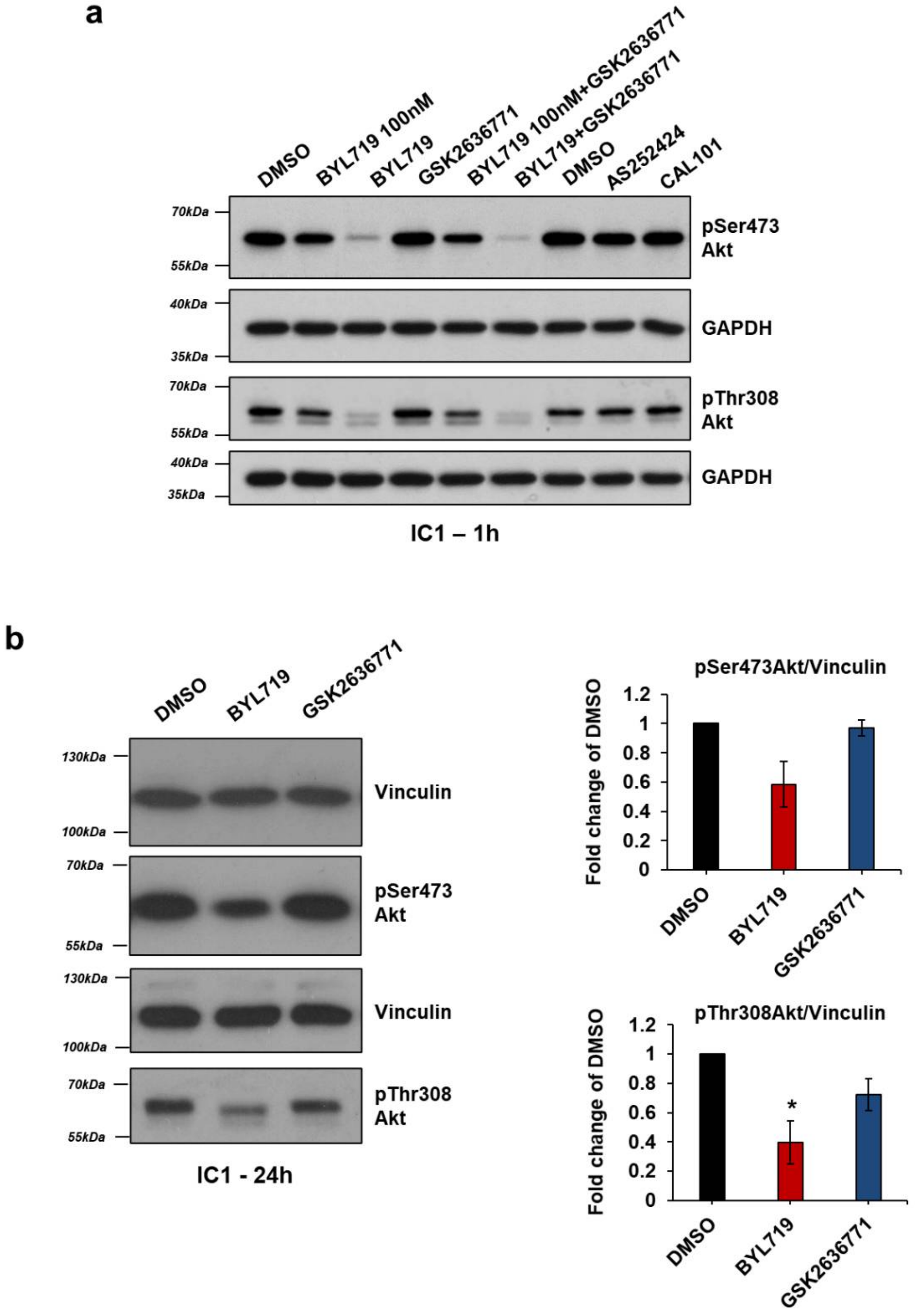

Supplementary Figure 14. BYL719 inhibits Akt activation in IC1 cells. IC1 cells were treated with the indicated inhibitors in complete medium supplemented with 10% FBS. Inhibitors were used at a final concentration of 1μM unless otherwise stated. Control cells were treated with DMSO. After 1h (**a**) or 24h (**b**), cells were lysed and analyzed by Western blotting using an anti pSer473 Akt or an anti pThr308 Akt antibody. Vinculin was used as loading control. Graphs in (**b**) represent results from densitometry analysis of phosphorylated Akt normalized to Vinculin levels. Data are expressed as fold change of results from control cells and are means ± SEM from n = 3 independent experiments (Student's t-Test, one-tailed distribution). * $p < 0.05$ vs. DMSO.
